# A Systematic Map of Genetic Variation in Plasmodium falciparum

**DOI:** 10.1371/journal.ppat.0020057

**Published:** 2006-06-23

**Authors:** Claire Kidgell, Sarah K Volkman, Johanna Daily, Justin O Borevitz, David Plouffe, Yingyao Zhou, Jeffrey R Johnson, Karine G. Le Roch, Ousmane Sarr, Omar Ndir, Soulyemane Mboup, Serge Batalov, Dyann F Wirth, Elizabeth A Winzeler

**Affiliations:** 1 Department of Cell Biology, The Scripps Research Institute, La Jolla, California, United States of America; 2 Department of Immunology and Infectious Diseases, Harvard School of Public Health, Boston, Massachusetts, United States of America; 3 Plant Biology Laboratory, Salk Institute for Biological Studies, La Jolla, California, United States of America; 4 Genomics Institute of the Novartis Research Foundation, San Diego, California, United States of America; 5 Faculty of Medicine and Pharmacy, Cheikh Anta Diop University, Dakar, Senegal; Northwestern University, United States of America

## Abstract

Discovering novel genes involved in immune evasion and drug resistance in the human malaria parasite, *Plasmodium falciparum,* is of critical importance to global health. Such knowledge may assist in the development of new effective vaccines and in the appropriate use of antimalarial drugs. By performing a full-genome scan of allelic variability in 14 field and laboratory strains of *P. falciparum,* we comprehensively identified ≈500 genes evolving at higher than neutral rates. The majority of the most variable genes have paralogs within the P. falciparum genome and may be subject to a different evolutionary clock than those without. The group of 211 variable genes without paralogs contains most known immunogens and a few drug targets, consistent with the idea that the human immune system and drug use is driving parasite evolution. We also reveal gene-amplification events including one surrounding *pfmdr1,* the P. falciparum multidrug-resistance gene, and a previously uncharacterized amplification centered around the P. falciparum GTP cyclohydrolase gene, the first enzyme in the folate biosynthesis pathway. Although GTP cyclohydrolase is not the known target of any current drugs, downstream members of the pathway are targeted by several widely used antimalarials. We speculate that an amplification of the GTP cyclohydrolase enzyme in the folate biosynthesis pathway may increase flux through this pathway and facilitate parasite resistance to antifolate drugs.

## Synopsis

Variability in the genome of the human malaria parasite, *Plasmodium falciparum,* is key to the parasite's ability to cause disease and overcome therapeutic interventions such as drugs and vaccines. Elucidating the extent of genetic variation in the malaria parasite will therefore be central to decreasing the malaria disease burden. The authors performed a full-genome scan of variability in different strains of P. falciparum and observed a nonrandom distribution of variation. In particular, those genes that are predicted to have roles in evading the host immune response or antimalarial drugs show significantly higher levels of variation. In addition, the authors speculate that a previously unreported genome amplification in the folate biosynthesis pathway correlates with resistance to the antimalarial drug sulfadoxine. Such data enable hypotheses to be made about the function of many of the unknown elements in the parasite's genome, which may permit the identification of new targets that can be investigated for incorporation into a malaria vaccine and may aid in the understanding of how the parasite withstands drug pressure.

## Introduction

Malaria is a significant burden on world health. The most severe form of human malaria is caused by the apicomplexan parasite *Plasmodium falciparum.* Genetic variation in this parasite is central to the pathogenesis of the organism, as allelic variability in different clones is thought to facilitate immune evasion [1]. This in turn has significantly impeded progress towards the development of an effective malaria vaccine [2]. Thus understanding the extent and basis of genetic variation is important for strategies of disease control. In addition, the study of genetic variation in different populations and clones that differ in susceptibility to drugs can reveal genes or genetic regions involved in drug resistance [3], and can thus facilitate the use of more appropriate therapies against P. falciparum and can save lives.

Despite a tradition of studying microsatellite-length polymorphisms in P. falciparum [4–7], many now feel that the most efficient way to study natural variation and population structure is by shotgun sequencing, single-nucleotide polymorphism (SNP) discovery [8], and genotyping using arrays or a variety of other high-throughput, low-cost approaches that are used in human genetics. These processes all have some limitations. First, sequencing is time-consuming, and even though the cost is falling, shotgun sequencing faces sampling issues and is ill suited to the task of finding genome duplications, deletions, and amplifications, some of which may have profound roles in drug resistance. While polony sequencing [9] or new methodologies [10] may eventually allow the genome of a malaria parasite to be fully sequenced in one day, at a reasonable cost, such sequencing methods are not yet in widespread use, and library construction generally remains a bottleneck in the production of sequence data. Furthermore, *P. falciparum-*genome intergenic regions are up to 90% AT rich and are known to recombine when cloned in other organisms; they created special challenges in the original sequencing of the genome [11]. Thus it is possible that the same low-cost approaches that can be applied to other microbial genomes may not work as well for *P. falciparum.* High-throughput genotyping of a subset of SNP markers discovered through the sequencing of laboratory strains is more efficient than sequencing a whole genome. However, this relies on the assumption that traits such as drug resistance will be transmitted throughout a population solely by meiotic recombination and not through the rapid emergence of novel alleles and haplotypes via mitotic recombination and mutation, which may occur in *P. falciparum.*

Allelic-variation scanning [12], which is a hybridization-based method for discovering and tracking natural variation, has the advantage that no libraries are required, sampling is consistent and is based on an array template, no primers need to be designed, and tens of thousands of genetic markers can be both discovered and typed in as little as one day in any parasite isolate, potentially using only a few milliliters of infected patient blood as starting material. While the nature of this technique dictates that all observed variation will be relative to the sequenced strain represented on the array, reproducibility is high and the method can be used for genotyping and mapping traits in genetic crosses or through the analysis of linkage disequilibrium and, as with full-genome sequencing, the discovery of genes under selection [13]. Most importantly, the method easily discovers genome-amplification events, which generally may not be readily revealed by even laborious full-genome sequencing. The simplicity of this approach could allow full-genome longitudinal studies of parasite variability to be performed on large numbers of samples in response to the introduction of a new drug or vaccine.

Here, we systematically sample genetic variation in the malaria parasite by analyzing the patterns that are derived from hybridizing genomic DNA to an oligonucleotide array covering approximately 50% of the coding regions of the P. falciparum genome [14]. We demonstrate a highly nonrandom distribution of variability in terms of functional classes of genes, and we reveal genes that appear to be under increased selection pressure from the host immune system. Understanding patterns of global natural variation in malaria parasites can also assist in the functional characterization of the genes encoded by the parasite's genome. Applying a “guilt by association” principle, we hypothesize that those highly variable, uncharacterized genes that show similar profiles of host selection, expression pattern, and localization to validated immunogens might make promising additional vaccine candidates for further investigation. We also report amplification events in a multidrug-resistance gene and describe a previously uncharacterized amplification in the GTP cyclohydrolase, the first enzyme in the frequently targeted folate biosynthesis pathway.

## Results

To investigate genome-wide diversity in the human malaria parasite in an unbiased, comparative fashion, genomic DNA from 14 different cloned P. falciparum lines, obtained from four different continents (Table 1), were labeled and hybridized in triplicate to a custom high-density oligonucleotide array [14] specifically designed for studying diversity in P. falciparum (see Materials and Methods, Table S1).

**Table 1:**
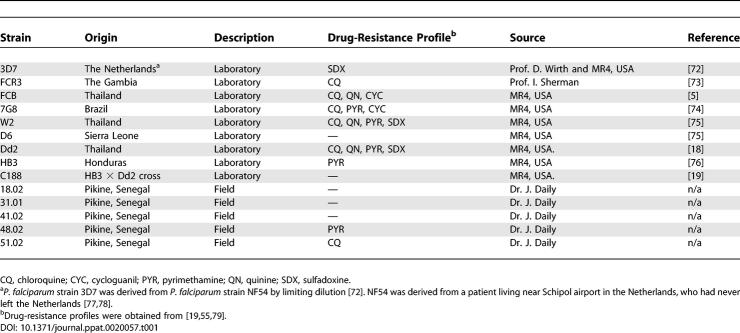
P. falciparum Strains Used in This Analysis

A single base change between a 25-mer microarray feature and its genomic target results in a reproducible loss of hybridization signal [12]. As the 25-mer probes used in this study were designed to map to unique locations in the genome, this approach enabled us to identify those genes that carry nucleotide mutations relative to the sequenced strain, 3D7. These single-feature polymorphisms (SFPs) are detected using an *F*-test on probe intensity values for three hybridizations for the query strain and the reference strain (3D7). Some of these SFPs correspond to SNPs which result in a change in protein structure, some to silent SNPs [15] and, in a few cases, these SFPs are the result of small insertion/deletion events (“indels”), which are more frequently observed in the non-coding regions [16], or in full deletions. We further required that the signal intensity for the 3D7 DNA be higher than that of the alternate strain since the physical location of gene duplications is unknown. To overcome any issues of variation acquired through long-term in vitro culture of laboratory strains, five of the 14 strains analyzed were isolated recently from Pikine, Senegal. These single clones, as indicated by genetic typing of the merozoite surface protein (MSP) 1 gene (unpublished data), were maintained in in vitro culture for only a short period.

Our analysis resulted in the identification of 23,653 SFPs across all strains for the 298,782 25-mer P. falciparum probes for the 23-Mb genome (Tables S2 and S3). The seven laboratory-derived strains displayed moderately higher numbers of polymorphic probes (Table S4) compared to the strains isolated from Senegal. However, the laboratory strain D6 (originally isolated from Sierra Leone) showed considerably fewer polymorphisms in comparison to all other strains analyzed.

**Figure 1:**
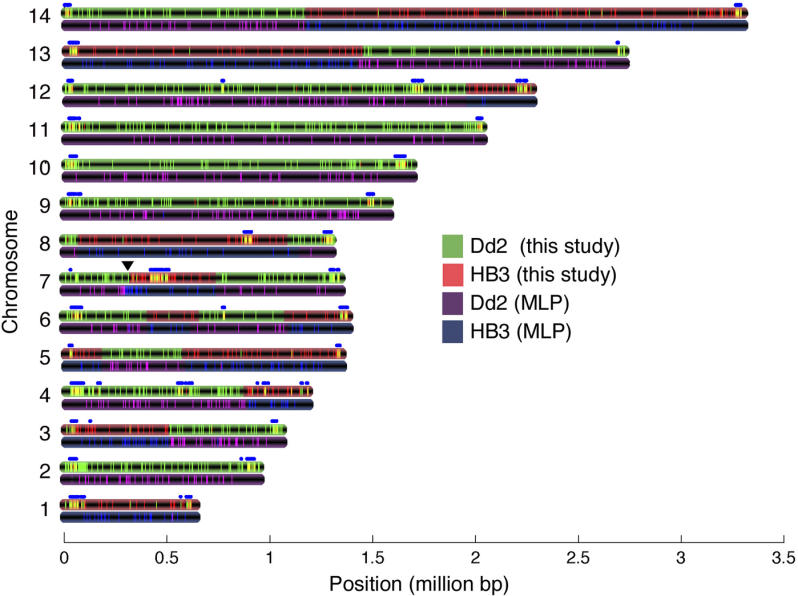
Mapping the Recombination Breakpoints across the 14 P. falciparum Chromosomes in the Chloroquine-Sensitive F1 Progeny Strain C188 A total of 14,289 SFPs were identified over three hybridizations by comparing HB3, Dd2, or the recombinant line C188 to 3D7. SFPs were recorded as HB3 (green bars) in the recombinant line if the probe was scored as polymorphic in both C188 and HB3 but not Dd2, or were recorded as Dd2 (red bars) if scored as polymorphic in Dd2 and C188 [19] but not HB3. Regions with a large number of markers scored as both HB3 and Dd2 are shown in yellow and are associated with the known locations of *var, rifin,* and *stevor* genes (shown as blue dots). Markers determined by microsatellite length mapping (MLP) are coloured purple and blue [7].

We had previously validated this method using a small Chromosome 2 microarray [17], and direct nucleotide sequencing of several alleles identified in this current study confirmed our accuracy with a low false-positive rate of <5% (Table 2). We also showed that these SFPs corresponded to heritable changes at specific chromosomal loci by examining their segregation patterns in a haploid progeny strain (C188) which was derived from a previous cross between a chloroquine-sensitive clone (HB3) and a chloroquine-resistant clone (Dd2) [18]. We identified recombination breakpoints that clearly corresponded to those previously published (Figure 1) [19], and the analysis of co-inheritance further confirmed a false-positive rate of less than 5%, although this number is likely to be inflated by the presumed existence of genuine mitotic gene-conversion events. As more genetic crosses between *Plasmodium* species become available, the rapid and reliable genotyping approach presented here will be an important tool for the mapping of multiple heritable traits, particularly in rodent malaria parasites [20], either individually or through bulk segregant analysis [21].

**Table 2:**
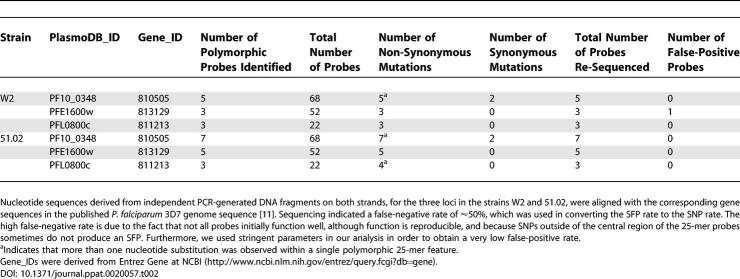
Direct Nucleotide Sequencing of Multiple Alleles across Three Loci in the P. falciparum Strains W2 and 51.02

**Figure 2:**
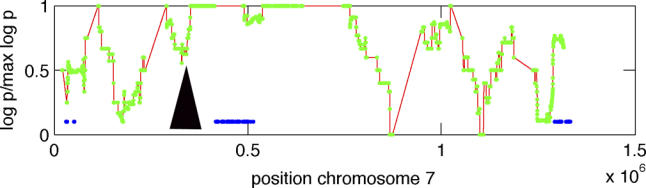
Linkage Disequilibrium surrounding *pfcrt (MAL7P1.27)* in FCB and Dd2 on Chromosome 7 Scores were generated by calculating the probability of observing the same genotype by chance over a moving 40-kbp window (with the probability of observing the same genotype for any one SFP by chance placed at 0.33). The plot shows the ratio between the probability and the maximum possible probability for regions with at least four SFPs, with 1 indicating the best possible score. The position of antigenic variation clusters *(vars, stevors,* or *rifins),* are shown in blue and are marked. SFPs mapping to these genes were excluded from the calculations because our data indicate that mitotic recombination may be occurring in these genes. *pfcrt,* which is located between bases 307,926 and 311,020 on Chromosome 7, is shown as a black triangle. The trough at *pfcrt* is likely to be due to the strong selective pressure on *pfcrt*, and is consistent with the observation that FCB and Dd2 have different alleles of *pfcrt* even though published SNP data also shows disequilibrium in surrounding regions [5]. Data for other chromosomes are shown in Figure S1.

It has been proposed that the analysis of linkage disequilibrium may permit the discovery of loci associated with drug resistance [4,5]. Previous linkage-disequilibrium analysis of 342 polymorphic microsatellite markers has shown that the mutations in the P. falciparum chloroquine-resistance transporter gene *(pfcrt) (MAL7P1.27),* which give rise to chloroquine resistance, have arisen on only four independent occasions and that these mutations have spread across the globe through selective sweeps [5]. We also observe that the SFP-derived haplotypes obtained from 4,369 SFPs distinguishing the chloroquine-resistant strains, FCB and Dd2, were nearly identical in the central region of Chromosome 7 and that this region (with the exception of the *PfEMP1* cluster) surrounds the *pfcrt* gene (Figure 2). This region is the only region that showed extensive disequilibrium and is accompanied by impressive probability scores. For example, the probability that the exact same haplotype would be observed for FCB and Dd2 by chance over 22 different SFPs in the region between bases 331,456 and 407,273 on Chromosome 7 is 1 in 3.1*E*−11. Disequilibrium was also noted at the same region in the comparison between FCR3 and Dd2, but was lacking in comparisons between other lines such as HB3 and Dd2. These data suggest that haplotype analysis of DNA derived from un-cloned drug-resistant and drug-sensitive parasites isolated directly from patients could indicate the chromosomal regions associated with drug resistance. In contrast to traditional mapping methods, this approach is not dependent on a set of pre-established markers or specialized primer sets. The entire SFP dataset (Tables S2 and S3), as well as a browsable interface showing the exact location of variation in each gene in the laboratory strains, can be viewed at http://carrier.gnf.org/publications/SFP.

### Classes of Non-Variable Genes

The ratio of SFPs to probes was highly variable depending on a gene's predicted function, chromosomal position, or life-cycle stage–expression [14]. Although the number of SFPs did not appear biased to any particular chromosome, an uneven distribution of polymorphic features within each chromosome was observed, as previously noted on Chromosome 2 [17]. Non-variable genes (more than ten probes, <10% SFP rate) accounted for 82% of the total genes located in the central regions of the chromosome (4,077/4,998 genes). In contrast 7,555 (≈75%) of the 10,122 probes in subtelomeric regions were polymorphic versus 12,978 (≈5%) of the remaining 247,910 probes (*p* ≤ 1.0*E*−100). Despite this observation, many genes that are located in the central regions of the chromosome show variability on an individual basis.

Low levels of variability were also found in different functional classes. For example, only 49 SFPs were observed for the 2,034 probes mapped to genes having a role in protein biosynthesis for the 13 strains interrogated. Assuming a false-negative SFP discovery rate of 50% and no linkage, this corresponds to one polymorphism for every ≈7,000 bases in each strain. In contrast, genes with putative roles or associated with immune evasion (*vars, rifins,* and *stevors* [22]) displayed a significantly higher estimated SNP rate of at least one polymorphism for every 200 bases (10,746 SFPs/13,018 probes), although this may be an underestimation as one probe may cover multiple SNPs. The fact that the majority of genes were exceptionally well conserved across strains supports the idea that the different strains of P. falciparum emerged through a population bottleneck so recently that multiple mutations have not accumulated [23,24].

Using previously published microarray data from strain 3D7, we found some correlation between the time of mRNA expression [14] and variability, with genes that are expressed during the parasite's brief extra-cellular phases (merozoite and sporozoite phases) showing almost twice the rate of variation (644 SFPs/8,368 probes for merozoite-expressed genes [*p* = 2.33*E*−46] and 1,043/5,396 for sporozoite-expressed genes [*p ≤* 1.0*E*−100]) as genes expressed during the late-trophozoite phase when DNA replication is occurring (373 SFPs/11,751 probes) (Table S5). The diversity observed within the genes expressed during these two extra-cellular life-cycle stages (merozoite and sporozoite stages) may result from an increased selection pressure from the host compared to those parasite stages that reside within the erythrocyte. Although we hypothesized that non-variable genes would show more reproducible expression profiles across strains and across various microarray-expression platforms, this was not the case. The average correlation coefficient for strains HB3 [25] and 3D7 [14] across expression profiles for expressed asexual erythrocytic stage genes, with SFP rates of less than 2% (more than nine probes), was 0.75 (*n* = 580), while for expressed variable genes (SFP rate >10%, more than nine probes) it was 0.70 (*n* = 99) (http://www.plasmodb.org) suggesting that expression patterns are generally not affected by variation. The greatest predictor of expression correlation across the strains and platforms was relative transcript abundance, with genes exhibiting low expression levels in the erythrocyte and high expression levels in the gametocyte or sporozoite stages (*n* = 394) showing the poorest correlation (0.28).

It has been suggested that intronic sequences are under neutral selection, and thus we calculated their SFP rates [26]. We observed a total of 309 SFPs/5,686 intronic probes, corresponding to a polymorphism for every ≈3,000 bases. However, 76/309 intronic SFPs are found within multi-gene families, while only 239/5,686 intronic probes are located in variable multi-gene families described below, suggesting that intronic sequences are not subject to neutrality when located in multi-gene families (*p* = 1.16*E*−32). This contrasts with the results of Castillo-Davis et al., who showed that intronic nucleotide substitution was not accelerated in duplicated genes in their comparison of the *P. yoelii yoelii* and P. falciparum genomes [26]. This discrepancy may be due to the fact that many members of P. falciparum multi-gene families do not have recognizable orthologs in the *P. yoelii yoelii* genome [27] and were excluded from the analysis. When intron sequences from multi-gene families are omitted, these findings suggest that members of housekeeping families, such as protein biosynthesis (49 SFPs/2,034 probes; SFP rate 2%), are evolving at less than the neutral rate (233 SFPs/5,447 probes; SFP rate 4%) (*p* = 0.000095).

### Types of Variable Genes

Approximately 10% (*n* = 457) of the genes in the malaria genome showed SFP rates (number of SFPs/total number of probes) of greater than 10% (with a minimum of ten probes)—more than twice the rate of neutrality—and thus could be classified as highly variable. These genes could be broken down into three broad categories, including members of multi-gene families, highly expressed immunogens (which are predicted to be under selection from the host immune system), and enzymes that may play a role in the parasite's response to drug pressure. Excluding the highly variable *var, rifin,* and *stevor* genes (*n* ≈ 290), known to be involved in antigenic variation, we identified a total of 234 variable genes, across all strains, with 49% (114/234 genes) containing a signal peptide (SP) or at least one transmembrane (TM) domain, suggesting that parasite-mediated exposure of proteins through externalization is important to the immune response. Of these 234 variable genes, 66 were annotated (Table 3) whereas 168 were classed as uncharacterized (Table S6), similar to that which was found in an analysis of SNPs on Chromosome 3 [8]. Within the individual strains, 128 variable genes were identified in the laboratory strains and 86 in the Senegal isolates (Tables S7 and S8). Interestingly, many of the best characterized P. falciparum genes (circumsporozoite [CS] protein, sporozoite surface protein 2, MSPs, erythrocyte-binding antigens) and genes associated with drug resistance, such as the P. falciparum chloroquine-resistance transporter*, pfcrt (MAL7P1.27),* reside within the highly variable, annotated genes that we identified (Table 3).

**Table 3:**
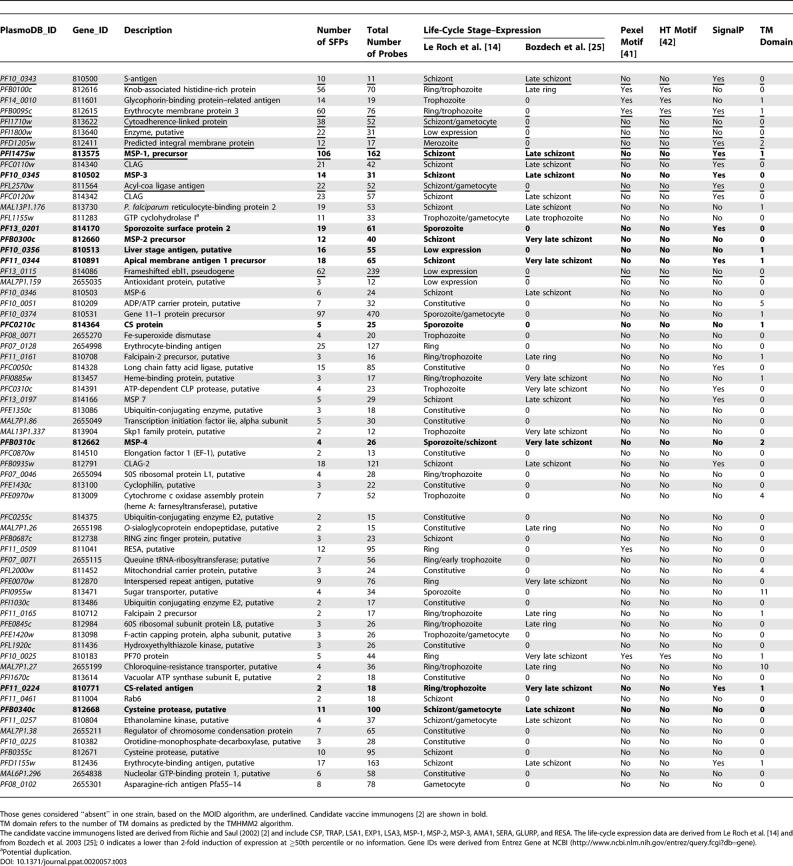
Highly Variable Annotated Genes (*n* = 66)

### Multi-Gene Families

We hypothesized that the presence of a paralog would predispose a gene to a loss of the 3D7 haplotype [28,29]. Gene-conversion events, which result in small rearrangements between different alleles or paralogs of the same gene, are the result of the cell's repair of a double-strand break (reviewed in [30]). In yeast, introducing a paralog can increase the rate of gene conversion [31]. In haploid, asexually replicating eukaryotic organisms such as P. falciparum or *Saccharomyces cerevisiae,* a double-strand break would likely result in cell death if the break occurred in a gene without a paralog that could be used in the repair process. However, if a paralogous gene were available for repair, the event would likely not be lethal, and evidence of the repair event would remain in the genome as a novel allele. It is also feasible that the presence of a paralog promotes higher rates of nonhomologous recombination [29]. Indeed, one of the strongest predictors of whether an uncharacterized gene would show excessive variability was whether or not it was assigned an ortholog/paralog group [32] (11,862 SFPs/23,781 probes for genes having a paralog versus 8,671 SFPs/234,251 probes for genes lacking a paralog (*p ≤* 1.0*E*−100). In fact, members of multi-gene families showed a 20-fold enrichment in the group of highly variable genes, relative to the genome as a whole, and a 5-fold increase (1,462 SFPs/11,048 probes; *p ≤* 1.0*E*−100) after members of the *var, rifin,* and *stevor* multi-gene families were excluded (10,746 SFPs/13,018 probes). While it may be possible that hybridization-based methods cannot be used to examine variability in multi-gene families, sequencing of *PF10_0348,* a member of the MSP family, and *PFE1600w,* a member of the ring-infected erythrocyte surface antigen (RESA) family, showed almost 100% fidelity in all polymorphisms scored (Table 2).

Although variation in *vars, stevors,* and *rifins* was expected, we identified other groups of consistently variable genes, such as one which contains 13 members (ortholog group 762970 [32]), almost all of which show extensive variation (in terms of SFPs) across the strains that we have examined (315 SFPs/1,051 probes). This gene group displays some homology to the P. knowlesi schizont-infected cell-agglutination variant antigen (SICA*var*) multi-gene family. All members (*n* = 13) of this gene family have previously been designated as the *surf* genes (surface-associated interspersed genes) [33], which encode a protein family, SURFINs, present on the surface of the infected erythrocyte and merozoite. The high degree of variability observed within these surface antigens implies that members of the *surf* gene family are likely to be evolving at higher rates [34] relative to the neutral polymorphism rate (30% versus 4%), which may also indicate a putative role for them in antigenic variation.

Other variable gene families include a recently identified multi-gene family called the Maurer's cleft proteins, which are trafficked to the surface of infected red cells [35] (ortholog group 765506 [32]) and showed variation at 29 of the 48 probes specific for this gene family. Some of the variable gene families may be related to human physiology: of the 12 genes that show an over-expression of mRNA transcripts in vivo compared to in vitro [36], nine are members of multi-gene families that show variation. One of these genes *(PF14_0752),* encodes an uncharacterized protein that is member of a 32-member multi-gene family (ortholog group 774486 [32]). A total of 91 SFPs/319 probes are variable within this ortholog group. Generally, a proportion of these multi-gene families show variation of mRNA expression of family members between different P. falciparum strains, suggesting that selective transcriptional silencing may further regulate their expression.

As previously noted, relatively conserved *var* genes have also been observed and are associated with binding to the host receptor, chondroitin sulfate A (CSA) [37–39]. Immunoepidemiologic evidence has suggested that this particular *var* gene variant is conserved between isolates and that it is implicated in malaria infection during pregnancy [37]. Of the polymorphism data available for approximately 70 of the annotated *var* genes, 17/70 genes showed significantly less variability (less than ten SFPs) than the remaining 53 genes, such as the previously characterized *var2CSA* gene *PFL0030c* [37]. Eleven out of 17 of these “conserved” *var* genes are annotated as truncated coding sequences, and one as a “pseudogene”. The inactivation of these coding sequences over time may be a cause or consequence of their specific role in malaria pathogenesis.

### Immunogens

Since certain blood-stage antigens of the malaria parasite, such as MSP-1 *(PFI1475w),* are highly expressed when the parasite invades the erythrocyte, they are likely to be under selection pressure from the host, and consequently are believed to display elevated rates of nucleotide substitution compared to the genome as a whole [40]. Such variable genes are thought to be the principal targets of protective immunity. To test the hypothesis that such genes would be under positive selection, we compared the SFP rate of probable immunogens to housekeeping genes. The SFP-to-probe ratio was significantly higher for characterized parasite immunogens in clinical development (CSP *[PFC0210c];* TRAP *[PF13_0201];* LSA1 *[PF10_0356];* LSA3 *[PFB0915w];* MSP-1 *[PFI1475w];* MSP-2 *[PFB0300c];* MSP-3 *[PF10_0345];* AMA1 *[PF11_0344];* SERA *[PFB0340c];* GLURP *[PF10_0344];* RESA *[PFA0110w];* and EXP1 *[PF11_0224]* [2] (220 SFPs/882 probes), compared with either housekeeping genes (such as those with roles in protein biosynthesis [49 SFPs/2,034 probes; *p* = 4.62*E*−76]) or compared with genes encoding proteins that are localized to the mitochondrion (114 SFPs/4,185 probes; *p* = 3.24*E*−94). Seven of these 11 characterized immunogens encode genes that are amongst the top 20 most highly variable, non-deleted annotated genes (Table 3); a ≈17-fold enrichment in the group of highly variable genes is shown relative to the genome as a whole. Between the 14 P. falciparum strains analyzed, strains Dd2 (Thailand), FCR3 (the Gambia), and FCB (Thailand) display the highest levels of polymorphisms in characterized parasite immunogens, while strains D6 (Sierra Leone) and 7G8 (Brazil) showed low levels of variation at these specific loci (Table S9). Overall, the laboratory strains show greater variation at these 11 loci compared to the Senegal strains, particularly at the MSP-1 locus (*PFI1475w*) (Table S9).

A conserved pentameric sequence (Pexel motif) [41] and an 11-amino acid signal (host-targeting [HT] motif) [42], which are frequently found in parasite proteins that are targeted to the red blood cell, have been identified, and such proteins have been proposed as candidates for proteins interacting with the host immune system. Excluding the highly variable *var, rifin,* and *stevor* genes, we observed a large enrichment for polymorphisms in genes for those proteins bearing a Pexel motif (609 SFPs/4,401 probes versus 9,178 SFPs/240,613 probes; *p* = 2.63*E*−157) or a HT motif (381 SFPs/2,983 probes versus 9,406 SFPs/242,031 probes; *p* = 7.75*E*−88) versus those that do not, although not all proteins bearing this signal show variation.

Based upon these observations, it is our hypothesis that many of the uncharacterized genes that show similar patterns of variability and gene expression to known antigens, such as MSP-1 *(PFI1475w)* or the CS protein *(PFC0210c),* also interact with the host immune system. Indeed, volunteers protected with an irradiated sporozoite vaccine all exhibited an immune response to the product of *PFL0800c* [43], a gene that shows a life cycle–dependent expression pattern that is similar to the well-characterized P. falciparum pre-erythrocytic stage antigen, the CS protein *(PFC0210c),* and which we demonstrate exhibits a comparable degree of variation (four SFPs/22 probes versus five SFPs/25 probes).

### Drug Resistance

While single point mutations in drug targets and receptors, which may not be identified by our analysis, can result in drug resistance, evidence suggests in some cases that multiple mutations are associated with resistance [44]. This may be because resistance mutations impose a fitness disadvantage and additional, compensatory mutations are required for resistance or to maintain a protein's 3D structure. It is known that the P. falciparum chloroquine-resistance transporter *(pfcrt) (MAL7P1.27)* shows a high rate of substitution with at least 15 different known haplotypes and 15 non-synonymous sites in a 424-amino acid protein [45]. In contrast, introns of housekeeping genes show low SNP rates (one SNP/27,000 bases) [23]. The list of highly variable genes (Table 3) includes the gene that is known to play a role in modulating antimalarial drug resistance, the P. falciparum chloroquine-resistance transporter *(pfcrt) (MAL7P1.27)* (four SFPs/36 probes; 11% SFP rate) [46]. The well-known drug-resistance gene dihydrofolate reductase *(dhfr) (PFD0830w)* [47], also shows an increased rate of variability (five SFPs/54 probes; 9% SFP rate) compared to the neutral rate (4%) [24].

As many antimalarials are derived from natural products that are likely to have off-target effects and to contribute to parasite toxicity, other enzymes that display variation (Table 3) may represent additional off-site targets. Such candidates potentially include heme A farnesyltransferase *(PFE0970w)* (seven SFPs/52 probes; 13 % SFP rate) or the heme-binding protein *(PFI0885w)* (three SFPs/17 probes; 17% SFP rate), which could be targeted by antimalarials that bind to heme derivatives, such as chloroquine and an ADP/ATP carrier protein *(PF10_0051)* (seven SFPs/32 probes; 21% SFP rate), which could modulate resistance to drugs targeting mitochondrial function, such as Atovaquone. A few non-enzymes that are not paralogs and do not contain a TM or SP domain were also variable. These non-SP/TM–containing proteins include the 50S *(PF07_0046)* and 60S *(PFE0845c)* ribosomal proteins. As many antibiotics target the ribosomal subunits, this variation may represent increased selection from indiscriminate antibiotic usage in the human host.

### Gene Amplifications

**Figure 3:**
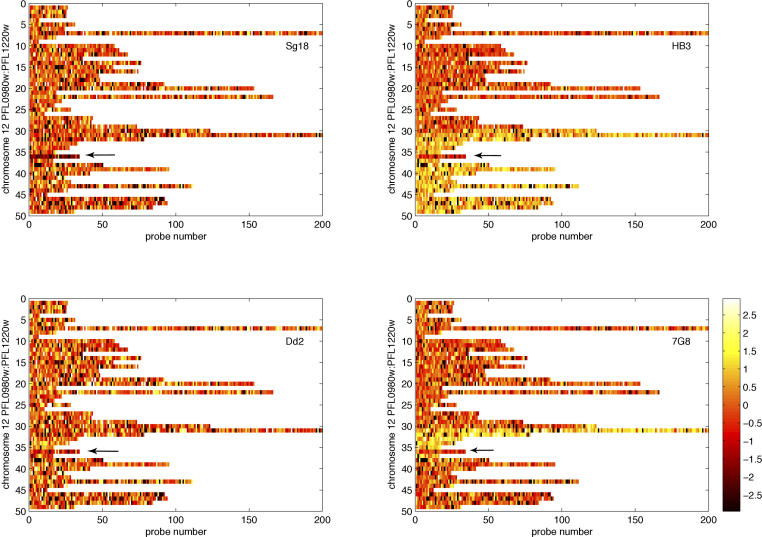
The Heat Map Shows the Base 2 Logarithm of the Ratio of the Normalized, Background Subtracted Probe Signal for the Listed Isolate (18.02, Dd2, 7G8 or HB3), Relative to the Sequenced Isolate, 3D7 All probes on the array were selected to be unique within 3D7. Each horizontal bar represents a single gene on the right arm of Chromosome 12. Dark probes, with low signal intensities, do not match any region in the genome of the listed isolate, while light probes, displaying high signal intensities, such as those near the GTP-cyclohydrolase gene *(PFL1155w)* in Dd2, HB3, and 7G8 contain matches to multiple regions of the genome. The signal intensity and the consecutive nature of the pattern suggest that strains 7G8, HB3, and Dd2 contain more than one copy of the GTP-cyclohydrolase gene *PFL1155w.* An arrow represents the location of the GTP-cyclohydrolase*PFL1155w* gene on Chromosome 12.

Notably absent from the list of highly variable genes (Table 3) was the P. falciparum multidrug-resistance gene, *pfmdr11 (PFE1150w),* which has been implicated in mefloquine resistance in both human [48] and rodent malaria infections [49]. This was not unexpected since it is amplifications of the *pfmdr1* gene that are associated with resistance, and we required all SFPs scored to show a higher signal in 3D7 relative to the alternate strain. Our analysis of hybridization ratios (see Materials and Methods) confirms the known *pfmdr1* amplification in the mefloquine-resistant strain Dd2 [50], and shows independent amplification events in strains FCR3 and FCB (Table 4). We are also able to detect further gene amplifications in other regions. For example, gene duplication is noted in the strain W2 encompassing the *PfRH1* gene *(PFD0110w)* on Chromosome 4 [51] and in the center of Chromosome 7. Our data identified an increased hybridization intensity surrounding the GTP cyclohydrolase gene *(PFL1155w)* on Chromosome 12 in seven out of the 14 strains analyzed (Table S10). GTP-cyclohydrolase encodes the first enzyme in the folate biosynthesis pathway. The folate pathway is the target of many antimalarials (Fansidar, WR99210, sulfadoxine, and pyrimethamine), chemotherapy agents (Methotrexate), and antibiotics (sulfa drugs). The mechanism of resistance to antifolates in vivo has been attributed to mutations within either the dihydrofolate reductase *(dhfr) (PFD0830w)* [47] or dihydropteroate synthase *(dhps) (PF08_0095)* genes [52], but mutations in the *dhps* gene may not always correlate with sulfadoxine resistance [53]. Although the regions surrounding the GTP cyclohydrolase gene *(PFL1155w)* appeared to be amplified, the median signal for all probes to GTP cyclohydrolase itself was actually significantly lower in the majority of strains relative to 3D7, suggesting an undiscovered amplification in this reference-sequenced strain (Figure 3).

**Table 4:**
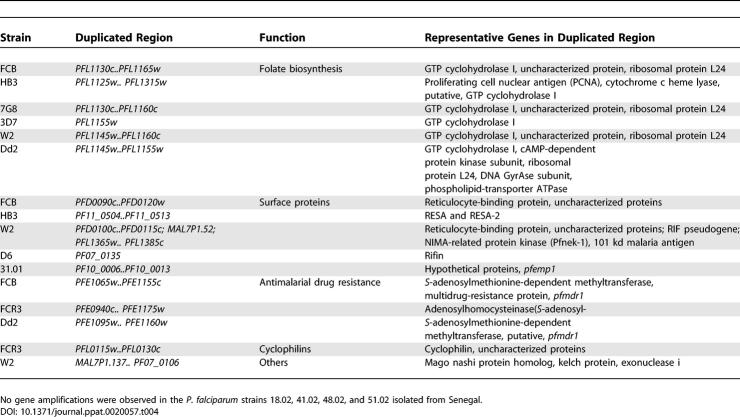
Description of the Genes Amplified in the P. falciparum Strains Analyzed

Real-time PCR analysis of the GTP-cyclohydrolase gene *(PFL1155w),* along with a β-tubulin control gene *(PF10_0084),* confirmed the independent amplification of this gene in 3D7 and in the P. falciparum strains W2, HB3, FCB, D6, 7G8, and Dd2 but not in FCR3 or any of the five strains isolated from Senegal (Table S11). Interestingly, it has been shown that the expression of bacterial GTP cyclohydrolase-1 in transgenic *Arabidopsis* results in a 1,250-fold and 2- to 4-fold enhancement in the levels of pterins and folates, respectively, suggesting that this is the rate-limiting enzyme in plants [54]. The presence of the amplification in most laboratory strains may indicate the importance of de novo folate biosynthesis in culture. However the absence of this amplification in the Senegal strains may also correlate with a lack of selection pressure in these recently acquired isolates, since antifolate drug regimes replaced chloroquine in Pikine, Senegal, only after the isolation of the strains used in this analysis. From the literature, it appears that this gene amplification correlates with the sulfadoxine-resistance profile previously published for particular strains analyzed [55–57]. We propose that partial sulfadoxine and/or pyrimethamine resistance may be linked to duplication of GTP cyclohydrolase *(PFL1155w),* which may permit an increased metabolic flux through the de novo pathway, although further investigation of a larger number of strains is required to confirm this association. Additional analysis of this duplication event from recently in vitro–adapted isolates in other geographic areas, as well as the continued analysis for the appearance of GTP-cyclohydrolase duplication in the Senegal strains over time (both in vivo and in vitro), will also be required.

### Gene Deletions

Previous studies have shown that arrays can be used to identify potential deletions using a comparative genome-hybridization approach that examines the ratios between the hybridization signals for two strains [25,58]. However, such a method makes it difficult to distinguish between a deletion and those cases where a gene is simply highly variable in one strain. The problem is compounded if only one or two probes per gene are used [25] versus the scores of probes used in these analyses. Here we use an algorithm, based on the match-only integral distribution (MOID) algorithm that returns a “present” call based on whether a probe-set distribution for a gene is similar to a series of background control probes [59] on the array. These control probes, which are not expected to hybridize to human or P. falciparum sequences, are predicted to have little signal associated with them and to show the same patterns as “deleted” genes. Analysis of background sequences indicated that they only had a 2% chance of being misclassified as “present”, and thus provides an estimate of the number of likely false positives.

**Figure 4:**
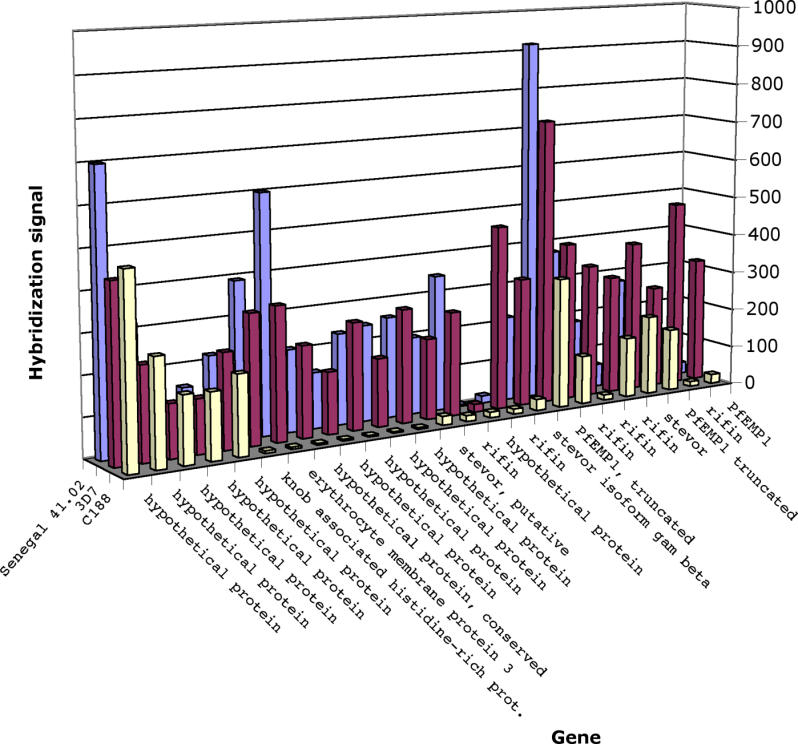
Example of a Deleted Region Hybridization levels were calculated with the MOID algorithm as previously described [59]. The figure shows the signal for 26 genes beginning with *PFB0010w* and ending with *PFB0120w* for three strains (41.02, 3D7, and C188). The background level (zero) was determined by analyzing the signal of non-P. falciparum genes on the array. Data for all genes and all strains are available from Table S12.

In applying this algorithm, every gene with at least six probes (*n* = 4,954) was considered present for 3D7 indicating a low false-negative rate as well, although gene-duplication events in strains other than 3D7 could mask the deletion of a very closely related gene (Table S12). While one might predict that highly variable genes might not be called “present,” the deletion analysis showed that hybridization to variable genes, even for the most variable *var, rifin,* and *stevor* genes, is generally higher than the background level (Figure 4; Table S9). For example, *PF10_0348* bears 23 SFPs/68 probes and is considered to be highly variable in our analysis. The MOID algorithm–derived *E* value for this gene ranged from 124 to 269 units with “present” *p*-values in the range of 1.0*E*−17 to 1.0*E*−47. In contrast, genes within the previously characterized 42-kbp deletion in P. falciparum strain W2 showed different patterns [17,60]. This deletion is likely to be associated with a loss of cytoadherence in parasite lines maintained in in vitro culture. The signal for *PfEMP3 (PFB0095c),* a gene in this region, ranged from 200–792 units in strains where it was considered present and from 18.1 to 4.6 units in strains where it was considered deleted. The *p*-values were in the range of 1.0*E*−222 for strains where it was considered present and from 1.0*E*−3.6 to 1.0*E*−1 where it was considered deleted. These differences are remarkable and allow a high degree of confidence in calling deletions.

We also identify deletions (Table S13) in several genes on the right arm of Chromosome 9 in strains FCR3 and FCB, including those encoding an enzyme *(PFI1800w),* an uncharacterized protein *(PFI1785w),* and the cytoadherence-linked asexual protein (CLAG, *PFI1710w*) [61]. The Maurer's cleft proteins [35], which co-localize with a 130-KDa membrane-associated protein found in both the Maurer's clefts and knobs, but only in a cytoadherent P. falciparum strain [62] (thus indicating a potential role in cytoadherence) were also often deleted. Despite the confidence that our method offers, difficulties in distinguishing a full deletion from a large partial deletion or substitution do exist, particularly for large genes such as MSP-1 *(PFI1475w).* The MSP-1 gene, to which there are 162 probes, was considered “absent” in one strain (HB3), although visual inspection of the gene showed a residual signal at the 3 prime end of the gene, consistent with the known 3 prime conservation of this well-studied immunogen. Because most other deleted genes were located in chromosomal clusters and were themselves adjacent to other deleted genes, which was not true for the S-antigen *(PF10_0343)* and MSP-1 *(PFI1475w),* our confidence in calling these genes “absent” is decreased. Efforts to PCR-amplify the entire 10-kbp MSP-1 region using primers outside of the variable regions were not successful.

## Discussion

Two recent studies examined selection in the P. falciparum genome: the first measured codon volatility [63], and the second identified orthologous gene sequences under selection through comparative sequencing of rodent parasite genomes [64]. A lack of correlation was observed between each gene's average SFP value, as determined by this study, and the corresponding *p-*value assigned by codon volatility (*r* = 0.06, Pearson's correlation) or the dN/dS ratio (*r* = 0.01, Pearson's correlation). However, because of the fundamental differences in the datasets and experimental approaches, we did not expect to observe a correlation. While the codon-volatility data measurement should be comparable, various groups have suggested that codon volatility makes unfounded assumptions about underlying mutational processes and is potentially not a robust approach to detect selection [65–67]. Secondly, Hall et al. [64] examined speciation events that occurred over a much larger time scale, while our data consider events that likely occurred more recently, potentially within the past 50 y. Furthermore, only 20% of the highly variable P. falciparum genes have detectable orthologs in the rodent malaria species, and only 13 out of the 77 genes showing a dN/dS ratio of >1 between P. berghei and P. chabaudi have an orthologous gene sequence present in P. falciparum (versus 70% of all genes). However, integrating the rodent dataset with our variability data may allow better prediction of potential drug-resistance genes. Characterized P. falciparum enzymes such as *dhfr,* orotidine monophosphate dehydrogenase, and heme A farnesyltransferase show very low dN/dS ratios but high rates of variability in our assay, suggesting roles in drug resistance.

These data may also be useful to systems biology research. Traditional gene-by-gene approaches to understanding gene function are difficult and time-consuming in *P. falciparum.* Systematic genome-wide analyses such as those described here can assist in the credentialing of genes to identify the most promising drug and vaccine targets. As with expression patterns, protein–protein interaction maps, or global-localization studies, the SFP rate offers an insight as to the potential function of uncharacterized genes; the SFP rate may indicate that a gene is likely to be involved in antigenic variation, that it is interacting with the host's immune system, or that it is evolving under drug pressure. Probes that tile through the P. falciparum genome can now be placed on a single microarray, which should permit the identification, at a higher resolution, of genes that are changing in response to the introduction of new drug treatment regimes, such as new artemisinin-based combination therapies [68]. Even if a single gene cannot be identified as being differentially under selection when comparing treated and untreated parasite populations, hybridization haplotypes associated with drug resistance [69,70] will likely be found. These data will also assist in the interpretation of traditional mapping data, because it is likely that SNPs in genes that are highly variable, or members of multi-gene families, will be less reliable than those that lie in genes that are largely monomorphic.

These data also shed light on the time of P. falciparum speciation. If one considers just the average number of SNPs [8] or SFPs, as in this study, the P. falciparum species appears ancient, but after excluding variation in genes that are likely to be evolving by mitotic recombination, the species looks considerably younger. While much additional work will be clearly required to confirm associations between mutations and resistance to individual drugs, such connections, if supported, will allow the design and implementation of more effective therapies. Ultimately, some of these genes involved in drug resistance, particularly those whose copy number is critical to parasite survival, may become the targets of novel drugs to treat malaria.

## Materials and Methods

### P. falciparum Affymetrix full-genome array.

Although useful for gene-expression analysis, the malaria full-genome oligonucleotide array [14] was specifically designed for studying diversity in *P. falciparum.* The design was optimized so as to produce as many nonoverlapping probes in coding regions as possible. The array contains 327,989 specific single-stranded 25-mer probes, of which 281,552 map to the exons of 5,179 genes that comprise ≈55% of the 23-Mbp genome. After excluding overlap between probes and repetitive regions (≈18% of the coding sequence), on average more than 53% of the exon sequences are covered by at least one probe. Probe coverage is significantly lower in the intragenic regions because of the frequency of repetitive sequences and the high AT content (up to 90%). Of the 327,989 single-stranded probes, 29,207 are perfect reverse complements of another. Because forward and reverse probes do not always exhibit identical hybridization behavior, the forward probe might be capable of revealing an SFP while its complement might not. Thus, reverse-complement probes were excluded from the SFP tally only if both the forward and the reverse complement detected an SFP. Reverse complements were excluded from the number of probes per genes listed in Tables 1–4.

### DNA methods.

P. falciparum parasites were cultured in leukocyte-depleted human O^+^ erythrocytes as previously described [71], and genomic DNA was isolated by standard phenol–chloroform extraction. RNA contamination was removed by treatment with RNAse at 37 °C for 1 h.

Genomic DNA (15 μg) from each P. falciparum strain (Table 1) and 2.5 ng each of Bio B, Bio C, Bio D, and Cre control plasmids were fragmented by treatment with DNase 1 and end-labeled with biotin [12]. Hybridization was subsequently carried out to the P. falciparum full-genome array [14] at 45 °C overnight, followed by a standard wash protocol and antibody staining (Affymetrix, Santa Clara, California, United States) and detection with streptavadin R-phycoerythrin conjugate and normal goat IgG. Arrays were scanned with an emission wavelength of 560 nm at 3-μm resolution using a confocal scanner (Affymetrix), and the signal intensity for each feature on the array was determined using the 70th percentile method in Microarray Suite 5 (Affymetrix). Three replicate hybridizations for each strain were carried out.

### Identification of SFPs.

SFPs were identified as described in Winzeler et al. [12]. Grids were aligned to the scanned images by the known feature dimensions of the array. The hybridization intensities for each of the elements in the grid were determined by the 75th percentile method in the Affymetrix GeneChip software package.

For each hybridization, an adjusted array-hybridization intensity value *(I)* was determined as the mean of the log signals of all features that showed minimal variation across all hybridizations. Then, for each feature on the array, a linear regression of the logarithm on *I* for all hybridizations was determined by the least-squares method, first under the null hypothesis that the reference (P. falciparum strain 3D7) and test P. falciparum strains had the same response and then under the alternative hypothesis that the reference (3D7) strain had a greater signal than the test strain. The models were compared with the *F-*test, and the same signal model was rejected in favor of a marker with 99% confidence.

### Gene amplifications and deletions.

Gene amplifications were identified by comparing an array hybridized with DNA for each strain to an array that had been hybridized with 3D7 DNA. Comparisons were made between arrays that appeared to be overall closest to one another in overall signal. As a first step, background was subtracted from probe signals, and overall array signals were normalized as previously described for the two arrays to be compared [14]. Background values were determined by analyzing nonhuman, non-P. falciparum control sequences on the array, also as previously described [14]. This step places the overall signal intensities of arrays in the same range as one another, but does not take into account the fact that strains that are different from the sequenced strain, 3D7, are expected to give an overall lower intensities because they contain SFPs. Thus, we computed an additional normalization factor, *N* (usually resulting in a 5% to 10% change). This was calculated by computing the log2 of the median signal ratio between the test strain and 3D7 for all probes to all genes that contained no SFPs in any of the strains analyzed (≈1,400 genes). In addition, probes with negative values in 3D7 or the test strain, or which showed log2 ratios of more than 4 or less than −4, were discarded.

The median value for all probe-sets was chosen as *N.* To identify duplications, all 3D7 probe values were multiplied by *N,* determined independently for each strain. Then the log2 of the ratio *(R)* of the test strain signal relative to *N*-normalized 3D7 was calculated for each probe in each probe-set. In order to eliminate probes complementary to SFPs or which could behave as outliers, for each probe-set, probes were discarded if *R* was greater than 4 or less than −4. A paired *t*-test was performed on the *R* values for the remaining probes in the probe-set using *p* ≤ 0.01. Next, we identified genes with a median *R* of greater than 0.7 for the remaining probes when the number of remaining probes was greater than six and the *t*-test indicated a likely difference between the intensities of the probe signal for 3D7 relative to test strain. The *t*-test was added in order to distinguish between a probe-set being amplified and a probe-set being polymorphic at only several probes. The value of 0.7 (roughly a 1.5-fold increase in signal) was determined empirically, and is slightly less than the expected 2-fold change because array probe signals are not expected to be perfectly linear with respect to copy number, and because the ratios for amplified genes are expected to show a distribution of values. A comparison of the HB3 and 3D7 ratios (Figure S2) shows a tight distribution around zero, as expected, with a secondary peak around 0.8. Genes with values of greater than 0.7 in the histogram are found exclusively in two chromosomal blocks; one surrounding GTP cyclohydrolase *(PFL1155w)* on Chromosome 12 and one on Chromosome 11 at the *PF10_0374* locus. However, while this method worked well for our data, it may not work as well for saturated arrays or low-intensity arrays. Potential duplications were visually examined to verify that the regions showed contiguous increases in signals. Short amplifications of small genes may have been missed.

To identify potential amplifications in 3D7, we compared the list of genes showing a 1.5-fold or greater change in 3D7 relative to each of the Senegal isolates (*n* = 5) as described above. This returned some genes that were likely to be highly polymorphic in individual Senegal strains, but such deletions were not held in common by all. Only two genes were shared by all the Senegal strains examined. These two genes were for GTP cyclohydrolase *(PFL1155w)* and P. falciparum 11-1 protein *PF10_0374,* the gene11-1 product, which is highly expressed during gametocytogenesis. Examination of the ratios for these genes (Table S7) is consistent with a 3D7 amplification (generally from 1.5- to 4-fold changes) rather than a Senegal deletion where ratios show a 20-fold difference in signal. Quantitative real-time PCR analysis further confirmed a probable amplification in 3D7 for GTP cyclohydrolase (Table S8).

In contrast, gene deletions were identified as follows. The custom-designed Affymetrix malaria full-genome array consists of 2,397 probes for 100 viral genes that serve as background controls [14]. Intensities from these probes represent the level of cross-hybridization for a deleted gene. A new probe-to-gene map was generated to include both sense and antisense probes, and the MOID algorithm [59] was applied to assign “present” and “absent” calls to each gene. Based on all the background control data collected in this study, this analysis, similar to that described in Le Roch et al. [14], shows that a deleted gene has only a 2% chance to be misclassified as “present” if it is required to have both an intensity level of *E* > 10 and a Kolmogorov–Smirnov test of log10P of less than −0.5. Excluding genes with less than six probes and the highly variable *var, rifin,* and *stevor* genes, we found a total of 33 genes being called “absent” in at least two out of the three hybridizations for each strain (Table S9).

### Mapping inheritable markers.

SFPs were identified in the strains Dd2, HB3, and C188, relative to 3D7 as described above*.* The inheritance of markers was determined by creating a matrix containing the SFP data for the two parental strains (Dd2 and HB3) and the progeny strain C188. The data were scored accordingly: if a marker is present in Dd2 and in the recombinant, but not in HB3, score as Dd2, but if a marker is in HB3 and the recombinant, but not Dd2, score as HB3. These data were then plotted relative to chromosome position, and subsequently potential recombination breakpoints could be clearly defined.

### Analysis of gene-expression data.

A tab-separated text file containing the correlation coefficient of expressed asexual erythrocytic genes across the P. falciparum strains 3D7, HB3, and Dd2, derived from the Affymetrix high-density oligonucleotide and glass-slide oligonucleotide microarray-expression platforms was obtained from PlasmoDB (http://www.plasmodb.org; Chris Stoeckert, personal communication). The average correlation coefficient of highly variable, expressed erythrocytic genes between strains 3D7 (Affymetrix platform, Winzeler laboratory) and strain HB3 (glass-slide platform, DeRisi laboratory) were derived.

### Real-time PCR analysis.

GTP-cyclohydrolase *(PFL1155w),* the uncharacterized protein *(PFL1145w),* ribosomal protein L24 *(PFL1150c),* and *pfmdr1 (PFE1150w)* copy number were assessed by real-time PCR amplification using the BioRad MyiQ apparatus (Hercules, California, United States) in the presence of SYBR-green. The following oligonucleotide primers were designed to specifically amplify each gene: PFL1155w-F 5′-AAACACCATCTTTTACCTTTTGAA-3′; PFL1155w-R 5′-AGCATCGTGCTCTTTAACTCC-3′; pfmdr1-F 5′-CAAGCGGAGTTTTTGCATTT-3′; pfmdr1-R 5′-TTGAGCGCTTTGACTGAATC-3′; β-tubulin-F 5′-TCGTCAACTTCCTTTGTGGA-3′; β-tubulin-R 5′-TCCCATTCCCACGTTTACAT-3′; PFL1145w-F 5′-GGGTATGCCCCTGGAAGTAT-3′; PFL1145w-R 5′-TCCACATGCTCCACCAATAA-3′; PFL1150c-F 5′-ACGCAAGAAATGCCTATTCA-3′; and PFL1150c-R 5′-CTTTGATGGGAGGCCCTATT-3′. Parallel amplification reactions were carried out in 96-well plates in 25 μl containing 0.5 μm of each forward and reverse primer, 1 μl of template DNA, and 12.5 μl of iQSYBR green PCR master mix (50 mM KCl, 20 mM Tris-HCl, 0.2 mM each dNTP [dATP, dCTP, dGTP, and dTTP], 0.6U iTaq DNA polymerase, 3 mM MgCl_2_, SYBR Green I, 10 nM fluorescein) (BioRad). Forty cycles were performed (95 °C 10 min, 95 °C 10 s, 55 °C 30 s, 60 °C 30 s) (Table S11).

### Nucleotide sequencing.

Oligonucleotide primers were designed to specifically amplify polymorphic regions of the *PF10_0348, PFL0800c,* and *PFE1600w* coding sequences (Table S14). Independent PCR-generated DNA fragments from both strands were sequenced using dRhodamine-labeled terminators. Nucleotide sequences were edited using the 4Peaks DNA analysis program v1.6 (http://www.mekentosj.com/4peaks).

### Statistical analysis.

The Fisher's exact test (http://www.matforsk.no/ola/fisher.htm) was used to determine the statistical relationship between each of the categorical variables in this analysis.

All the P. falciparum 3D7 genome data and gene annotations used for analysis in the work described in this paper are available for download at http://www.plasmodb.org. The gene-identification numbers (Gene_ID) were derived from Entrez Gene at NCBI (http://www.ncbi.nlm.nih.gov/entrez/query.fcgi?db=gene).

## Supporting Information

Figure S1 Linkage Disequilibrium Analysis Shown is the linkage disequilibrium analysis of SFP-derived haplotypes obtained from 4,369 SFPs distinguishing the chloroquine resistant strains FCB and Dd2, across the 14 P. falciparum chromosomes. Scores were generated by calculating the probability of observing the same genotype by chance over a moving 40 kb window (with the probability of observing the same genotype for any one SFP by chance placed at 0.33). The plot shows the ratio between the probability and the maximum possible probability for regions with at least four SFPs with 1 indicating the best possible score. The position of antigenic variation clusters (*vars*, *stevors* or *rifins*), are shown in blue and are marked. SFPs mapping to these genes were excluded from the calculations because our data indicates that mitotic recombination may be occurring in these genes. *pfcrt*, which is located between bases 307,926 and 311,020 on chromosome 7 is shown as a black triangle. A: Chromosomes 1–7, B: 8–14.
(2.4 MB PDF)

Figure S2 A Ratio Histogram of the HB3/3D7 Signal Intensities As expected, a tight distribution around zero is observed, with a secondary peak around 0.8, for 4,300 genes with more than six probes, excluding the *var, rifin,* and *stevor* genes. Genes with log2 ratios of greater than 0.7, and classed as gene amplifications in HB3, are found exclusively in two chromosomal blocks: one on Chromosome 12 surrounding the GTP-cyclohydrolase gene *(PFL1155w)* and one on Chromosome 11, encompassing the region from *PF11_0504* to *PF11_0513.*
(22 KB TIF)

Table S1 Probe File (38Mb) for the SCRmalaria Array This file contains data for 327,989 P. falciparum-specific 25 mer probes selected for use in this analysis. Probes located in both the coding and non-coding sequences from both strands are included. Non-specific and un-mapped probes have been removed. This file does not contain any probes from the Plasmodium yoelii sequence. Affymetrix standard control probes and probes from human and mouse are marked as “controls” in the “Reporter Usage” column. Generic mismatches are marked as “background” in the “Reporter Usage” column.
(6.7 MB ZIP)

Table S2 SFP Data by Strain This tab-separated file shows the strain hybridization pattern for every P. falciparum isolate for the 23,653 SFPs.
(3.2 MB TXT)

Table S3 SFP Data by Gene This tab-separated text file shows SFP data compiled by gene.
(257 KB TXT)

Table S4 Polymorphism Variation across the P. falciparum Strains Analyzed (55 KB PDF)

Table S5 The Number of Polymorphic Probes per Cluster of Genes Expressed at Each Stage of the P. falciparum Life Cycle Clusters 1–15, along with representative genes for each cluster, are further described in Le Roch et al. [14]. Essentially, genes were grouped into clusters, based on the expression correlation throughout the P. falciparum life cycle, by a robust *k*-means program. While many of the genes belonging to each cluster encode proteins with unknown function, those genes with defined cellular roles permitted a life-cycle stage to be attributed to each of the 15 clusters defined. Virtually all known sporozoite-specific genes, such as the CS protein, reside within cluster 1, and most genes that have known roles in invasion, such as the MSPs, are found in cluster 15, validating the cluster assignments.
(57 KB PDF)

Table S6 Highly Variable, Uncharacterized Genes (*n* = 168) Genes that are likely to be deleted are underlined.
(73 KB PDF)

Table S7 A List of the Highly Variable, Annotated Genes Observed in the Laboratory Strains (*n* = 34) Aside from those deleted genes (underlined), no distinct differences were observed between the classes of highly variable genes within the laboratory strains compared to the Senegal strains (Table 5). Indeed, no significant differences were observed in the variation within immunogens (*p* = 0.87) and protein biosynthesis (*p* = 0.45) genes between the two groups of isolates, but variation in multi-gene families was significant (*p* = 6.37*E*−58).
(66 KB PDF)

Table S8 A List of the Highly Variable, Annotated Genes in the P. falciparum Strains from Senegal (*n* = 25) The notably higher variation observed in the Senegal strains at the glycophorin-binding protein–related antigen *(PF14_0010)* locus is due to a deletion of this gene (underlined) in strain 51.02.
(64 KB PDF)

Table S9 Variation in Characterized Parasite Immunogens in Clinical Development Candidate parasite immunogens were derived from Richie and Saul [2].
(56 KB PDF)

Table S10 Predicted Copy Number for Amplified Genes The log2 (Ratio) and copy number, for all P. falciparum Strains, of the GTP-Cyclohydrolase I *(PFL1155w),* the two flanking genes *(PFL1150c* and *PFL1160w),* as well as an uncharacterized control gene *(PFL0650c),* also located on Chromosome 12. The ratio and copy number of the P. falciparum multidrug-resistance protein, *pfmdr1 (PFE1150w)* and the P. falciparum 11-1 protein *(PF10_0374)* are also represented. A ratio histogram of the log2 (ratio) for the HB3/3D7 signal intensity (Figure S1) verifies the thresholds used to identify gene amplifications. The GTP-cyclohydrolase copy numbers were extrapolated based on three copies in 3D7, and gene *pf11-1* was extrapolated based on two copies.
(56 KB PDF)

Table S11 Threshold Cycle Values Derived from the Real-Time PCR Amplification of the P. falciparum Genes *(PFL1155w, PFL1145w, PFL1150c,* and *PFE1150w),* along with a β-Tubulin Control Gene *(PF10_0084)*
A parallel reaction was carried out for each primer pair, and the mean threshold cycle (Ct) value and standard deviation are presented.
(58 KB PDF)

Table S12 Gene Deletions Results of the MOID Analysis Showing Genes Detected as “Present” (8.9 MB XLS)

Table S13 Description of the Genes Deleted in the P. falciparum Strains Analyzed; Deletions Are Reported Only for Genes with ≥ Six Probes No deletions in strain D6 were observed.
(75 KB PDF)

Table S14 Oligonucleotide Primer Sequences (54 KB PDF)

## Abbreviations

CLAG: cytoadherence-linked asexual protein
CS: circumsporozoite
CSA: chondroitin sulfate A
HT: host-targeting
MOID: match-only integral distribution
MSP: merozoite surface protein
RESA: ring-infected erythrocyte surface antigen
SFP: single-feature polymorphism
SNP: single-nucleotide polymorphism
SP: signal peptide
TM: transmembrane
