# Correction: A Systematic Map of Genetic Variation in Plasmodium falciparum

**DOI:** 10.1371/journal.ppat.0020096

**Published:** 2006-08-25

**Authors:** Claire Kidgell, Sarah K Volkman, Johanna Daily, Justin O Borevitz, David Plouffe, Yingyao Zhou, Jeffrey R Johnson, Karine G. Le Roch, Ousmane Sarr, Omar Ndir, Soulyemane Mboup, Serge Batalov, Dyann F Wirth, Elizabeth A Winzeler

In *PLoS Pathogens*, volume 2, issue 6: DOI: 10.1371/journal.ppat.0020057

In the Materials and Methods section, the following Supporting Information files were mislabeled: Table S7 should be Table S10, Table S8 should be Table S11, and Table S9 should be Table S12. The corrected citations in text follow:

To identify potential amplifications in 3D7, we compared the list of genes showing a 1.5-fold or greater change in 3D7 relative to each of the Senegal isolates (*n* = 5) as described above. This returned some genes that were likely to be highly polymorphic in individual Senegal strains, but such deletions were not held in common by all. Only two genes were shared by all the Senegal strains examined. These two genes were for GTP cyclohydrolase (PFL1155w) and P. falciparum 11–1 protein PF10_0374, the gene11–1 product, which is highly expressed during gametocytogenesis. Examination of the ratios for these genes (Table S10) is consistent with a 3D7 amplification (generally from 1.5- to 4-fold changes) rather than a Senegal deletion where ratios show a 20-fold difference in signal. Quantitative real-time PCR analysis further confirmed a probable amplification in 3D7 for GTP cyclohydrolase (Table S11).

In contrast, gene deletions were identified as follows. The custom-designed Affymetrix malaria full-genome array consists of 2,397 probes for 100 viral genes that serve as background controls [14]. Intensities from these probes represent the level of cross-hybridization for a deleted gene. A new probe-to-gene map was generated to include both sense and antisense probes, and the MOID algorithm [59] was applied to assign “present” and “absent” calls to each gene. Based on all the background control data collected in this study, this analysis, similar to that described in Le Roch et al. [14], shows that a deleted gene has only a 2% chance to be misclassified as “present” if it is required to have both an intensity level of E > 10 and a Kolmogorov-Smirnov test of log10P of less than −0.5. Excluding genes with fewer than six probes and the highly variable *var, rifin,* and *stevor* genes, we found a total of 33 genes being called “absent” in at least two out of the three hybridizations for each strain (Table S12).

Additionally, the Supporting Information file in the legend text for Table S7 was mislabeled. Table 5 should be Table S8. The corrected citation in text follows:

Aside from those deleted genes (underlined), no distinct differences were observed between the classes of highly variable genes within the laboratory strains compared with the Senegal strains (Table S8). Indeed, no significant differences were observed in the variation within immunogens (*p* = 0.87) and protein biosynthesis (*p* = 0.45) genes between the two groups of isolates, but variation in multi-gene families was significant (*p* = 6.37*E*−58).

