# Supplementary material for: A Systematic Map of Genetic Variation in Plasmodium falciparum
Source: PLoS Pathog. 2006 Jun 23;2(6):e57. doi: 10.1371/journal.ppat.0020057 (PMC1480597; doi:10.1371/journal.ppat.0020057)
Supplement: Supplementary file 1 [file ppat.0020057.sg001.pdf]

A

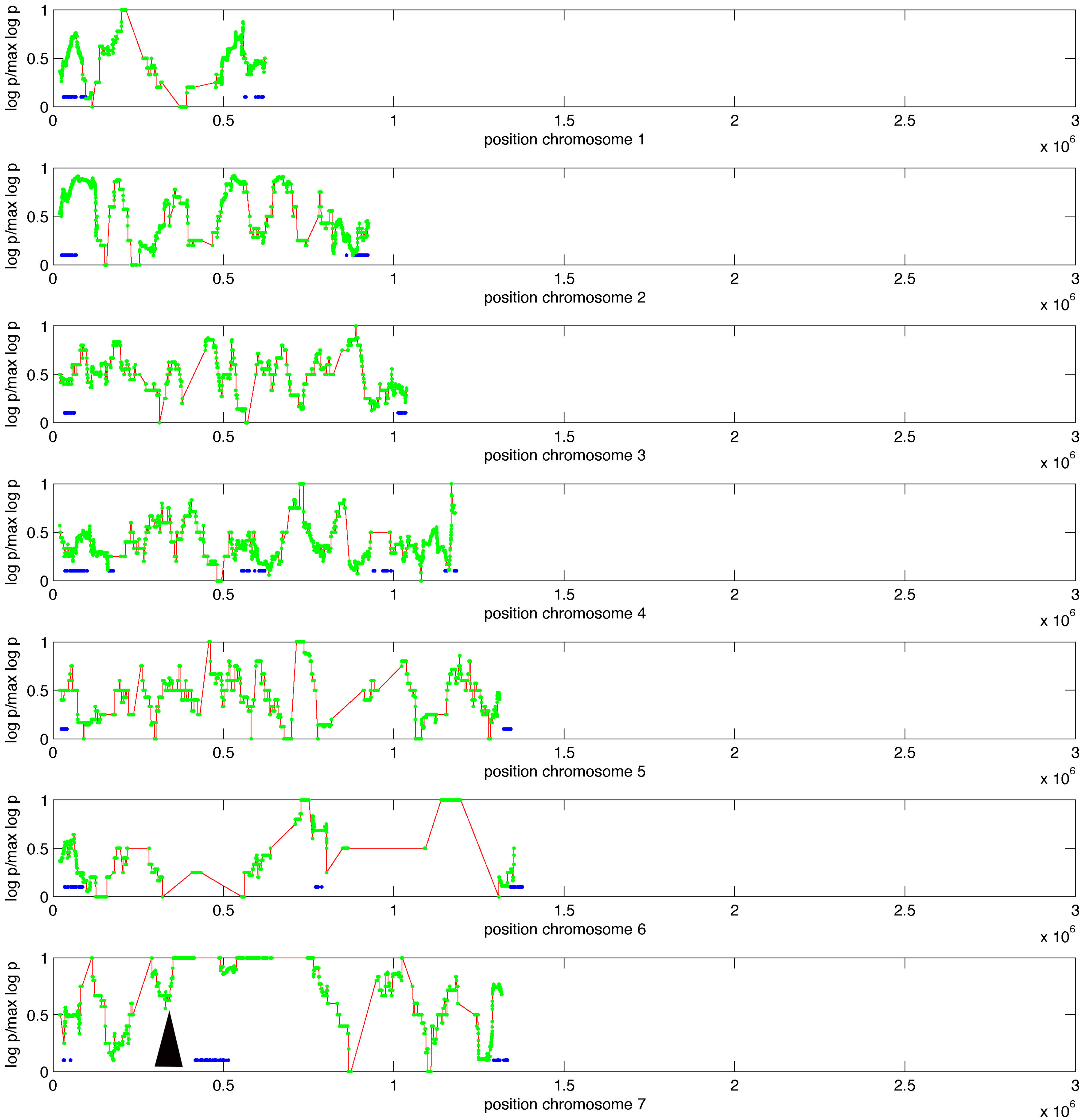

log p/max log p
position chromosome 1
position chromosome 2
position chromosome 3
position chromosome 4
position chromosome 5
position chromosome 6
position chromosome 7
x 10^6

**B**

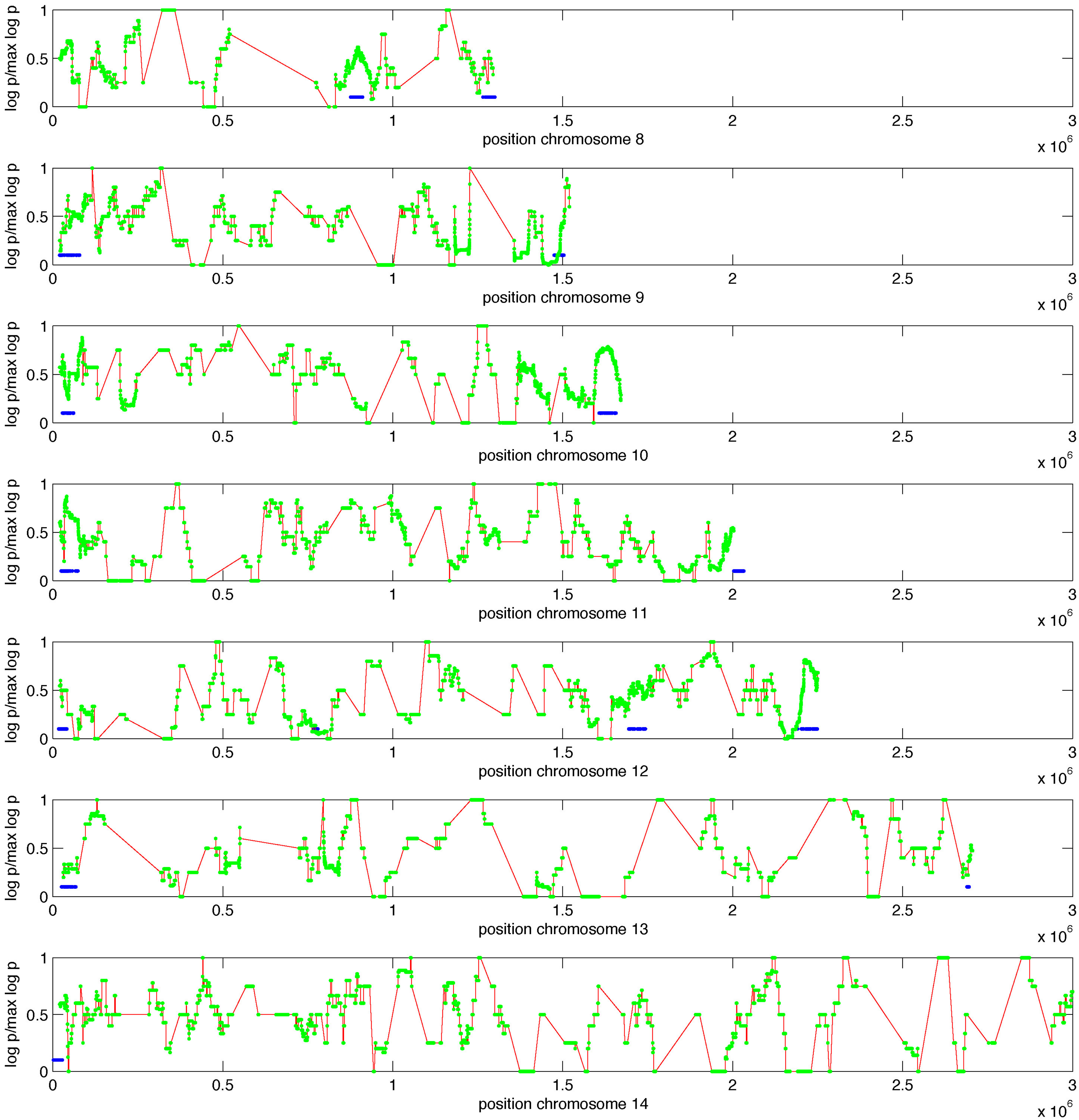

log p/max log p
position chromosome 8
position chromosome 9
position chromosome 10
position chromosome 11
position chromosome 12
position chromosome 13
position chromosome 14
0
0.5
1
1.5
2
2.5
3
x 10^6
