# Supplementary material for: A Systematic Map of Genetic Variation in Plasmodium falciparum
Source: PLoS Pathog. 2006 Jun 23;2(6):e57. doi: 10.1371/journal.ppat.0020057 (PMC1480597; doi:10.1371/journal.ppat.0020057)
Supplement: Supplementary file 2 [file ppat.0020057.st004.pdf]

Supplemental Table S4 Polymorphism variation across the *P. falciparum* strains analyzed.

Strain	Origin	Total number of SFPs	SFPs detected in characterized genes (total number of probes; 92,974)	SFPs detected in uncharacterized genes (total number of probes; 165,058)	Candidate Vaccine antigens* (total number of probes; 882)
7G8	Brazil	8920	7198	1068	37
D6	Sierra Leone	2721	2007	504	22
W2	Thailand	10621	6809	2813	65
HB3	Honduras	10550	7967	1751	141
Dd2	Thailand	9736	7466	1450	69
FCR3	The Gambia	10549	7826	1818	129
FCB	Thailand	9457	7245	1425	130
18.02	Pikine, Senegal	7536	6089	795	41
31.01	Pikine, Senegal	8505	6740	1014	53
41.02	Pikine, Senegal	8739	6729	1302	44
48.02	Pikine, Senegal	7932	5989	1207	41
51.02	Pikine, Senegal	11787	8642	2008	67
C188	HB3 x Dd2 cross	9714	7446	1468	66

Footnote. * The candidate vaccine antigens are described in Richie and Saul (2002) [2] and include CSP, TRAP, LSA1, LSA3, MSP-1, MSP-2, MSP-3, AMA-1, SERA, GLURP, EXP1 and RESA.
