# Supplementary material for: A Systematic Map of Genetic Variation in Plasmodium falciparum
Source: PLoS Pathog. 2006 Jun 23;2(6):e57. doi: 10.1371/journal.ppat.0020057 (PMC1480597; doi:10.1371/journal.ppat.0020057)
Supplement: Supplementary file 3 [file ppat.0020057.st005.pdf]

Supplemental Table S5 The number of polymorphic probes per cluster of genes expressed at each stage of the *P. falciparum* life cycle. Clusters 1 -15, along with representative genes for each cluster are further described in Le Roch et al. 2003. Essentially, genes were grouped into clusters, based on the expression correlation throughout the *P. falciparum* life cycle, by a robust *k*-means program. While many of the genes belonging to each cluster encode proteins with unknown function, those genes with defined cellular roles permitted a life cycle stage to be attributed to each of the 15 clusters defined. Virtually all known sporozoite-specific genes, such as the circumsporozoite (CS) protein reside within cluster 1 and most genes that have known roles in invasion, such as the merozoite surface proteins (MSPs) are found in cluster 15, validating the cluster assignments.

Cluster	Total number of genes represented by each cluster	Average number of probes per gene, per cluster	Time of peak expression (representative genes and function)	Number of SFPs	Total number of probes
1	105	51.3	Sporozoite (immune evasion)	1043	5396
2	152	52.9	Sporozoite / gametocyte (cell cycle regulation)	377	8046
3	218	48.1	Gametocyte (meiosis)	375	10494
4	95	47.4	Ring (fatty acid metabolism, early transcribed membrane proteins)	380	4509
5	109	41.3	Ring / early trophozoite (protein synthesis)	328	6507
6	167	55.4	Ring / trophozoite (transcription)	359	9258
7	93	43.5	Ring / trophozoite (ribosomal proteins, hexose metabolism, knob associated histidine rich protein, trophozoite antigens)	281	4050
8	125	46.4	Trophozoite/ gametocyte (immune evasion: maurer's cleft proteins)	322	5800
9	120	37.5	Trophozoite/ gametocyte (cell-cycle regulation)	130	4510
10	225	57.1	Trophozoite (DNA replication/mitotic cell cycle)	430	12866
11	110	65.1	Trophozoite (DNA replication/mitotic cell cycle)	236	7170
12	238	49.3	Trophozoite (DNA replication/mitotic cell cycle)	373	11751
13	180	68.4	Schizont / gametocyte (DNA replication)	484	12329
14	163	68.8	Sporozoite / schizont / gametocyte (actin filament-based processes)	541	11216
15	130	64.3	Schizont (invasion)	644	8368
