# Supplementary material for: A Systematic Map of Genetic Variation in Plasmodium falciparum
Source: PLoS Pathog. 2006 Jun 23;2(6):e57. doi: 10.1371/journal.ppat.0020057 (PMC1480597; doi:10.1371/journal.ppat.0020057)
Supplement: Supplementary file 4 [file ppat.0020057.st006.pdf]

Supplemental Table S6 Highly variable, uncharacterized genes (n=168). Genes that are likely to be deleted are underlined.

PlasmoDB_ID	Gene_ID	Number of SFPs	Total number of probes	Life cycle stage expression		Pexel motif[1]	HT motif[2]	SignalP	TM domain
				Le Roch et al [b]	Bozdech et al [c]				
PF14_0734	812316	20	20	Ring / Trophozoite	0	No	No	No	2
PF14_0732	812314	38	44	Sporozoite / Schizont	Late schizont	Yes	No	No	1
PF14_0733	812315	23	27	Low expression	Late ring	No	Yes	No	1
PFB0070w	812610	10	12	Low expression	0	Yes	Yes	Yes	2
PFB0080c	812612	27	33	Ring / Trophozoite	Late ring	Yes	No	No	1
PFI1785w	813637	27	33	Low expression	0	Yes	Yes	No	0
PFB0090c	812614	35	43	Ring / Trophozoite	Late ring	Yes	No	No	0
PFB0075c	812611	17	22	Trophozoite / Gametocyte	0	Yes	Yes	No	2
PF11_0012	810580	8	11	Low expression	0	No	No	No	0
PF10_0014	810172	11	16	Ring	0	No	No	No	0
PFB0085c	812613	57	85	Ring	Very late schizont	Yes	No	No	1
PFA0650w	813265	62	93	Schizont	0	No	No	No	1
PFC0045w	814327	8	13	Low expression	0	Yes	Yes	No	0
PFD1215w	812407	11	18	Low expression	0	Yes	No	No	1
PFD0105c	812528	44	75	Schizont	Late schizont	No	No	No	1
MAL6P1.21	2654746	10	18	Low expression	0	Yes	Yes	No	0
MAL7P1.58	2655005	6	12	Low expression	0	No	No	No	2
PF10_0355	810512	31	65	Low expression	0	No	No	No	1
PFL2565w	811563	9	19	Ring	Early ring	Yes	Yes	Yes	0
PF07_0004	2654971	28	69	Ring	Very late schizont	No	Yes	No	3
PF14_0747	812329	38	95	Low expression	0	No	No	No	1
PF11_0042	810595	4	11	Low expression	0	No	No	No	1
PFA0665w	813269	71	196	Low expression	Very late schizont	No	No	No	0
PF14_0726	812308	11	31	Sporozoite	0	No	No	No	1
MAL8P1.66	2655392	7	20	Low expression	0	No	No	Yes	1
PF10_0348	810505	23	68	Schizont	Late schizont	No	No	Yes	0
MAL8P1.162	2655360	6	19	Low expression	0	No	No	No	0
PFA0725w	813281	5	16	Low expression	0	No	No	No	1
MAL8P1.1	2655214	15	48	Low expression	0	No	No	No	1
PF10_0342	810499	13	45	Sporozoite / Schizont	Late schizont	No	No	Yes	0
PF10_0257	810414	4	14	Low expression	Late ring	No	No	No	1
PFD1160w	812428	46	164	Low expression	0	No	No	No	0
MAL7P1.6	2655007	5	18	Ring	0	No	Yes	No	0
PFL2555w	811561	5	18	Low expression	0	Yes	No	No	1
PF08_0002	2655451	37	153	Low expression	0	No	No	No	1
PF13_0074	814052	35	145	Low expression	0	No	No	No	2
PFB0970c	812798	5	21	Low expression	Very late schizont	Yes	Yes	Yes	0
PFA0100c	813157	3	13	Low expression	0	No	No	No	0
PFB0950w	812793	6	27	Low expression	0	No	No	No	3
MAL6P1.262	2654801	4	18	Low expression	0	No	No	No	0
PFD0090c	812529	3	15	Trophozoite / Gametocyte	Late trophozoite	No	No	No	1

PlasmoDB_ID	Gene_ID	Number of SFPs	Total number of probes	Life cycle stage expression		Pexel motif[1]	HT motif[2]	SignalP	TM domain
				Le Roch et al [b]	Bozdech et al [c]				
MAL7P1.59	2655006	3	15	Low expression	0	No	No	No	0
PF14_0739	812321	3	15	Low expression	0	No	No	Yes	0
PFA0205w	813178	11	56	Sporozoite / Gametocyte	Late schizont	No	No	No	5
PFD0595w	812584	10	52	Schizont / Gametocyte	Late schizont	No	No	No	2
PF11_0513	811045	10	52	Ring	Very late schizont	Yes	No	No	0
PFE1595c	813128	4	21	Low expression	0	Yes	No	No	1
PFI1780w	813636	5	27	Ring / Trophozoite	Late ring	No	No	No	1
PFB0965c	812797	2	11	Low expression	0	No	No	No	0
MAL7P1.128	2655151	2	11	Low expression	0	No	No	No	1
PFI0800c	813440	2	11	Sporozoite	Low expression	No	No	No	4
PFL0800c	811213	4	22	Low expression	Very late schizont	No	No	Yes	0
PFL1225c	811297	2	11	Low expression	Late ring	No	No	No	0
PF13_0203	814172	2	11	Low expression	0	No	No	Yes	0
PF14_0030	811612	4	22	Sporozoite/gametocyte	0	No	No	No	0
PF07_0042	2655090	20	112	Gametocyte	Late trophozoite	No	No	No	0
PFE0890c	812993	3	17	Gametocyte	0	No	No	No	0
PFL1440c	811340	3	17	Low expression	0	No	No	No	1
PF14_0727	812309	12	68	Low expression	0	No	No	No	0
PFA0655w	813267	9	52	Low expression	0	No	No	No	0
MAL7P1.32	2655205	5	29	Low expression	0	No	No	Yes	1
PF10_0281	810438	6	35	Sporozoite / Schizont	Late schizont	No	No	Yes	1
PFD0075w	812533	5	30	Low expression	Late ring	No	No	No	1
PFE0790c	812972	2	12	Low expression	0	No	No	No	0
MAL7P1.160	2655037	2	12	Low expression	0	No	No	No	0
MAL8P1.6	2655342	3	18	Sporozoite	0	No	No	No	3
PF10_0193	810351	2	12	Low expression	0	No	No	No	0
MAL13P1.111	813659	3	18	Low expression	0	No	No	Yes	0
MAL13P1.113	813661	2	12	Low expression	0	No	No	No	0
PF14_0452	812034	2	12	Sporozoite	0	No	No	No	0
PF14_0467	812049	4	24	Low expression	0	No	No	Yes	0
MAL8P1.62	2655388	3	19	Schizont	Late schizont	No	No	No	0
PF08_0005	2655454	4	26	Sporozoite	0	No	No	Yes	1
PF11_0511	811043	2	13	Low expression	Late ring	No	No	No	0
MAL13P1.42	813931	4	26	Trophozoite	Late schizont	No	No	No	1
PF14_0019	811592	4	26	Low expression	0	Yes	No	No	0
PFI0500w	813380	4	27	Low expression	0	No	No	Yes	1
PF10_0350	810507	7	48	Ring / Trophozoite	Late ring	No	No	Yes	0
PF11_0271	810818	11	76	Low expression	0	No	No	No	0
PFB0140w	812624	2	14	Low expression	0	No	No	No	3
PFD0660w	812576	3	21	Schizont/ Gametocyte	Late schizont	No	No	No	1
PFD1185w	812425	3	21	Low expression	Late ring	Yes	No	No	1
PF07_0052	2655096	2	14	Low expression	0	No	No	No	0
MAL8P1.160	2655358	2	14	Low expression	0	No	No	No	2
MAL13P1.233	813792	2	14	Low expression	Late ring	No	No	No	0
PF10_0244	810401	8	57	Low expression	Late schizont	No	No	No	0

PlasmoDB_ID	Gene_ID	Number of SFPs	Total number of probes	Life cycle stage expression		Pexel motif[1]	HT motif[2]	SignalP	TM domain
				Le Roch et al [b]	Bozdech et al [c]				
MAL6P1.209	2654745	7	50	Low expression	0	No	No	No	0
PFB0610c	812722	4	29	Low expression	0	No	No	No	0
MAL6P1.63	2654889	4	29	Low expression	0	No	No	No	0
PFD0705c	812565	3	22	Low expression	Late schizont	No	No	No	0
PF08_0101	2655300	8	59	Low expression	0	No	No	No	0
PF08_0015	2655464	2	15	Gametocyte	0	No	No	Yes	1
PF13_0194	814164	2	15	Ring	Early ring	No	No	Yes	1
PFD0610w	812582	4	31	Low expression	0	No	No	No	0
PF14_0596	812178	6	47	Low expression	0	No	No	No	6
PFA0625w	813260	12	95	Low expression	0	No	No	No	1
PFA0670c	813270	3	24	Ring	0	Yes	Yes	No	3
PF10_0164	810322	2	16	Trophozoite	Very late schizont	No	No	Yes	2
PF10_0280	810437	2	16	Low expression	Late schizont	No	No	No	0
PF11_0281	810828	3	24	Low expression	0	No	No	No	0
PFL1805c	811413	2	16	Low expression	0	No	No	No	0
MAL13P1.129	813679	2	16	Low expression	0	No	No	No	1
MAL13P1.268	813829	5	40	Ring	Early ring	No	No	No	0
PF11_0036	810589	6	49	Trophozoite	0	No	No	No	0
PFE0395c	812868	4	33	Sporozoite	Very late schizont	No	No	Yes	1
PFE1025c	813020	3	25	Trophozoite	0	No	No	No	0
PFL0990w	811250	3	25	Low expression	0	No	No	Yes	3
PFC0460w	814425	2	17	Low expression	0	No	No	No	2
PF07_0083	2655127	2	17	Low expression	0	No	No	No	0
PFI0725c	813425	2	17	Low expression	Very late schizont	No	No	No	0
PF10_0341	810498	8	68	Low expression	0	No	No	No	0
PF11_0409	810955	2	17	Low expression	Late ring	No	No	No	0
PF13_0127	814098	4	34	Schizont	Late schizont	No	No	No	0
PF13_0348	814307	8	68	Ring / Trophozoite	0	No	No	No	0
PF14_0138	811719	2	17	Trophozoite	Late schizont	No	No	No	0
PFA0165c	813170	7	60	Low expression	0	No	No	No	2
PFB0105c	812617	3	26	Ring	Very late schizont	Yes	No	No	1
PFE1600w	813129	6	52	Ring / Trophozoite	0	Yes	No	No	0
PFD1145c	812437	4	35	Schizont	Very late schizont	No	No	No	0
PF14_0087	811668	4	35	Trophozoite	Late ring	No	No	No	0
PFE0985w	813012	6	53	Ring / Trophozoite	0	No	No	No	0
PFA0715c	813279	2	18	Low exrpession	0	Yes	No	Yes	3
PFD1085w	812440	2	18	Low expression	0	No	No	No	0
PFE0755c	812965	3	27	Low expression	0	No	No	No	5
PFE1460w	813106	2	18	Trophozoite	0	No	No	Yes	0
MAL6P1.196	2654731	2	18	Schizont / Gametocyte	0	No	No	No	0
PF08_0024	2655474	3	27	Gametocyte	0	No	No	Yes	0
MAL8P1.41	2655382	2	18	Low expression	0	No	No	No	1
PF08_0073	2655272	4	36	Sporozoite / Gametocyte	0	No	No	No	0
MAL8P1.99	2655450	2	18	Trophozoite	0	No	No	No	0

PlasmoDB_ID Gene_ID Number of Total number Life cycle stage expression Pexel motif[1] HT motif[2] SignalP TM domain

		SFPs	of probes	Le Roch et al [b]	Bozdech et al [c]				
PFI0810c	813442	2	18	Trophozoite	0	No	No	No	0
PF10_0042	810200	2	18	Low expression	0	No	No	No	0
PF10_0055	810213	2	18	Low expression	0	No	No	No	1
PF11_0476	811019	2	18	Low expression	0	No	No	No	0
PFL0855c	811223	4	36	Low expression	0	No	No	No	0
PF14_0394	811976	2	18	Low expression	0	No	No	No	1
PF14_0752	812333	3	27	Low expression	Late ring	Yes	No	No	1
PF10_0206	810363	5	46	Low expression	0	No	No	No	0
PF11_0462	811005	4	37	Low expression	0	No	No	No	0
PFL0865w	811225	4	37	Trophozoite	0	No	No	No	0
PF13_0181	814151	4	37	Sporozoite	0	No	No	No	0
PF13_0264	814229	4	37	Schizont / Gametocyte	0	No	No	No	0
PFC0570c	814450	3	28	Low expression	0	No	No	No	0
PF10_0068	810226	3	28	Ring / trophozoite	Late trophozoite	No	No	No	0
PFL1485w	811349	3	28	Ring / Trophozoite	0	No	No	No	0
MAL6P1.268	2654807	5	47	Low expression	0	No	No	No	0
PFE0360c	812879	7	66	Sporozoite	Late schizont	No	No	No	0
PFA0230c	813183	2	19	Low expression	0	No	No	No	0
PFC0095c	814337	2	19	Low expression	0	No	No	No	1
PFD0605c	812586	2	19	Low expression	0	No	No	No	0
PF08_0084	2655283	2	19	Low expression	0	No	No	No	0
PF10_0336	810493	2	19	Schizont / Gametocyte	Very late schizont	No	No	No	0
PFL0775w	811208	2	19	Trophozoite	0	No	No	No	5
PFL2020c	811456	4	38	Low expression	0	No	No	Yes	1
PF14_0269	811851	2	19	Low expression	0	No	No	No	0
PF13_0103	814079	5	48	Gametocyte	0	No	No	No	0
PF08_0137	2655336	8	77	Ring / Trophozoite	Late ring	No	No	No	1
MAL6P1.267	2654806	9	87	Low expression	0	No	No	No	0
MAL8P1.63	2655389	3	29	Trophozoite / Gametocyte	0	No	No	No	1
PF10_0161	810319	6	58	Low expression	0	No	No	No	0
PFL1760w	811404	3	29	Ring / Trophozoite	0	No	No	No	0
MAL13P1.285	813847	3	29	Low expression	Late schizont	No	No	No	1
PF14_0387	811969	3	29	Trophozoite	Late trophozoite	No	No	No	11
PFC1011c	814541	4	39	Low expression	0	No	No	No	6
PF13_0343	814302	4	39	Low expression	0	No	No	No	0
PFL1800w	811412	9	88	Ring / Trophozoite	0	No	No	No	2
MAL13P1.114	813662	19	186	Low expression	Late schizont	No	No	No	1
PF10_0195	810353	5	49	Gametocyte	0	No	No	No	3

Footnotes

1 Marti et al., 2004 [41]

2 Hiller et al., 2004 [42]

TM domain Number of transmembrane domains as predicted by the TMHMM2 algorithm. The life cycle expression data is derived from Le Roch et al. 2003 [14] [b] and Bozdech et al. 2003 [25] [c]; 0 indicates a lower than 2-fold induction of expression at > = 50th percentile or no information. Gene IDs were derived from Entrez Gene at NCBI (http://www.ncbi.nlm.nih.gov/entrez/query.fcgi?db=gene).
