# Supplementary material for: A Systematic Map of Genetic Variation in Plasmodium falciparum
Source: PLoS Pathog. 2006 Jun 23;2(6):e57. doi: 10.1371/journal.ppat.0020057 (PMC1480597; doi:10.1371/journal.ppat.0020057)
Supplement: Supplementary file 5 [file ppat.0020057.st007.pdf]

Supplemental Table S7 A list of the highly variable, annotated genes observed in the laboratory strains (n = 34). Aside from those deleted genes (underlined), no distinct differences were observed between the classes of highly variable genes within the laboratory strains compared to the Senegal strains (**Supplemental Table S8**). Indeed, no significant differences were observed in the variation within immunogens (*P* = 0.87) and protein biosynthesis (*P* = 0.45) genes, between the two groups of isolates but variation in multi-gene families was significant (*P* = 6.37E-58).

Laboratory

PlasmoDB_ID	Gene_ID	Description	Number of SFPs	Total number of probes	Life cycle stage expression		Pexel motif[1]	HT motif[2]	SignalP	TM domain	Highly variable in Senegal strains
					Le Roch et al.[b]	Bozdech et al. [c]					
PFB0100c	812616	Knob associated histidine-rich protein	55	70	Ring / Trophozoite	Late ring	Yes	Yes	No	0	No
PFI1710w	813622	Cytoadherence-linked protein	38	52	Schizont / Gametocyte	0	No	No	No	0	No
PF10_0343	810500	S-antigen	8	11	Schizont	Late schizont	No	No	Yes	0	Yes
PFI1800w	813640	Enzyme, putative	22	31	Low expression	0	No	No	No	0	No
PFI1475w	813575	Merozoite surface protein 1, precursor	98	162	Schizont	Late schizont	No	No	Yes	1	Yes
PFL2570w	811564	Acyl-coa ligase antigen	22	52	Schizont / Gametocyte	0	No	No	Yes	0	No
PFD1205w	812411	Predicted integral membrane protein	7	17	Merozoite	0	No	No	Yes	2	Yes
MAL13P1.176	813730	Plasmodium falciparum reticulocyte binding protein 2 homolog b	19	53	Schizont	Late schizont	No	No	No	1	No
PF10_0345	810502	Merozoite surface protein-3	9	31	Schizont	Late schizont	No	No	Yes	0	Yes
PF10_0346	810503	Merozoite surface protein 6	6	24	Schizont	Late schizont	No	No	No	0	No
PF13_0115	814086	Frameshifted ebl1, pseudogene	56	239	Low expression	0	No	No	No	0	No
PFC0120w	814342	Cytoadherence linked asexual protein, CLAG	12	57	Schizont	Late schizont	No	No	Yes	0	No
PF11_0344	810891	Apical membrane antigen 1 precursor	13	65	Schizont	Very late schizont	No	No	Yes	1	Yes
PF08_0071	2655270	Fe-superoxide dismutase	4	20	Trophozoite	0	No	No	No	0	No
PFC0110w	814340	Cytoadherence linked asexual protein, CLAG	8	42	Schizont	Late schizont	No	No	Yes	0	Yes
PF07_0128	2654998	Erythrocyte binding antigen	24	127	Ring	0	No	No	No	0	Yes
PFC0310c	814391	ATP-dependent CLP protease, putative	4	23	Trophozoite	Very late schizont	No	No	Yes	0	Yes
MAL13P1.337	813904	Skp1 family protein, putative	2	12	Trophozoite	Very late schizont	No	No	No	0	No
PFE1350c	813086	Ubiquitin-conjugating enzyme, putative	3	18	Trophozoite / Schizont	0	No	No	No	0	No

PlasmoDB_ID	Gene_ID	Description	Number of SFPs	Total number of probes	Life cycle stage expression		Pexel motif[1]	HT motif[2]	SignalP	TM domains	Highly variable in Senegal strains
					Le Roch et al. [b]	Bozdech et al. [c]					
PF10_0356	810513	Liver stage antigen, putative	9	55	Low expression	0	No	No	No	1	Yes
PF14_0010	811601	Glycophorin binding protein-related antigen	3	19	Trophozoite	0	Yes	Yes	No	1	Yes
PFC0050c	814328	Long chain fatty acid ligase, putative	12	85	Constitutive	0	No	No	Yes	0	No
PF13_0197	814166	Merozoite surface protein 7	4	29	Schizont	Late schizont	No	No	Yes	0	Yes
PF13_0201	814170	Sporozoite surface protein 2	8	61	Sporozoite	0	No	No	Yes	0	Yes
PFB0687c	812738	RING zinc finger protein, putative	3	23	Schizont	0	No	No	No	0	No
PF11_0161	810708	Falcipain-2 precursor, putative	2	16	Ring / Trophozoite	Late ring	No	No	No	1	No
PFC0210c	814364	Circumsporozoite (CS) protein	3	25	Sporozoite	0	No	No	No	1	Yes
PF11_0165	810712	Falcipain 2 precursor	2	17	Ring / Trophozoite	Late ring	No	No	No	1	No
PFB0935w	812791	Cytoadherence linked asexual protein 2	14	121	Schizont	Late schizont	No	No	Yes	0	Yes
PFE0845c	812984	60S ribosomal subunit protein L8, putative	3	26	Ring / Trophozoite	Late ring	No	No	No	0	No
PFE1420w	813098	F-actin capping protein, alpha subunit, putative	3	26	Trophozoite / Gametocyte	0	No	No	No	0	No
MAL7P1.27	2655199	Chloroquine resistance transporter, putative	4	36	Ring / Trophozoite	Late ring	No	No	No	10	No
PF11_0224	810771	Circumsporozoite-related antigen	2	18	Ring / Trophozoite	Very late schizont	No	No	Yes	1	No
PFI1670c	813614	Vacuolar ATP synthase subunit E, putative	2	18	Constitutive	0	No	No	No	0	No

Footnote.

1 Marti et al. 2004 [41]

2 Hiller et al., 2004 [42]

TM domain Number of transmembrane domains as predicted by the TMHMM2 algorithm.

The life cycle expression data is derived from Le Roch et al. 2003 [14] [b] and Bozdech et al. 2003 [25] [c]; 0 indicates a lower than 2-fold induction of expression at > = 50th percentile or no information. Gene IDs were derived from Entrez Gene at NCBI (http://www.ncbi.nlm.nih.gov/entrez/query.fcgi?db=gene).
