# Supplementary material for: A Systematic Map of Genetic Variation in Plasmodium falciparum
Source: PLoS Pathog. 2006 Jun 23;2(6):e57. doi: 10.1371/journal.ppat.0020057 (PMC1480597; doi:10.1371/journal.ppat.0020057)
Supplement: Supplementary file 6 [file ppat.0020057.st008.pdf]

Supplemental Table S8 A list of the highly variable, annotated genes in the *P. falciparum* strains from Senegal (n = 25). The notably higher variation observed in the Senegal strains at the glycophorin binding protein-related antigen (PF14_0010) locus is due to a deletion of this gene (underlined) in strain 51.02.

Senegal

PlasmoDB_ID	Gene_ID	Description	Number of SFPs	Total number of probes	Life cycle stage expression: Le Roch et al [b]	Life cycle stage expression: Bozdech et al [c]	Pexel motif[1]	HT motif[2]	SignalP	TM domain	Highly variable in laboratory strains
PF10_0343	810500	S-antigen	10	11	Schizont	Late schizont	No	No	Yes	0	Yes
PF14_0010	811601	Glycophorin binding protein-related antigen	14	19	Trophozoite	0	Yes	Yes	No	1	Yes
PFD1205w	812411	Predicted integral membrane protein	9	17	Merozoite	0	No	No	Yes	2	Yes
PFC0110w	814340	Cytoadherence linked asexual protein, CLAG	20	42	Schizont	Late schizont	No	No	Yes	0	Yes
PF10_0345	810502	Merozoite surface protein-3	12	31	Schizont	Late schizont	No	No	Yes	0	Yes
PFC0120w	814342	Cytoadherence linked asexual protein, CLAG	21	57	Schizont	Late schizont	No	No	Yes	0	Yes
PFL1155w	811283	GTP cyclohydrolase I	9	33	Trophozoite / Gametocyte	Late trophozoite	No	No	No	0	No
PF10_0356	810513	Liver stage antigen, putative	15	55	Low expression	0	No	No	No	1	Yes
PFB0100c	812616	Knob associated histidine-rich protein	19	70	Ring / Trophozoite	Late ring	Yes	Yes	No	0	Yes
PF13_0201	814170	Sporozoite surface protein 2	13	61	Sporozoite	0	No	No	Yes	0	Yes
PF10_0051	810209	ADP/ATP carrier protein, putative	6	32	Constitutive	0	No	No	No	5	No
PF11_0344	810891	Apical membrane antigen 1 precursor	12	65	Schizont	Very late schizont	No	No	Yes	1	Yes
PFI0885w	813457	Heme binding protein, putative	3	17	Ring / Trophozoite	Very late schizont	No	No	No	1	No
PF13_0197	814166	Merozoite surface protein 7	5	29	Schizont	Late schizont	No	No	Yes	0	Yes
MAL7P1.159	2655035	Antioxidant protein, putative	2	12	Low expression	0	No	No	No	0	No
PF10_0374	810531	Gene 11-1 protein precursor	77	470	Sporozoite / Gametocyte	0	No	No	No	1	No
PF07_0128	2654998	Erythrocyte binding antigen	19	127	Ring	0	No	No	No	0	Yes
PF07_0071	2655115	Queuine tRNA-ribosyltransferase; putative	7	56	Ring / Early trophozoite	0	No	No	No	0	No
PFC0210c	814364	Circumsporozoite (CS) protein	3	25	Sporozoite	0	No	No	No	1	Yes
PFE0070w	812870	Interspersed repeat antigen, putative	9	76	Ring	Very late schizont	No	No	No	0	No
PFB0310c	812662	Merozoite surface protein 4	3	26	Sporozoite / Schizont	Very late schizont	No	No	No	2	No
PFI1475w	813575	Merozoite surface protein 1, precursor	18	162	Schizont	Late schizont	No	No	Yes	1	Yes
PFB0935w	812791	Cytoadherence linked asexual protein 2	13	121	Schizont	Late schizont	No	No	Yes	0	Yes

PlasmoDB_ID	Gene_ID	Description	Number of SFPs	Total number of probes	Life cycle stage expression		Pexel motif[1]	HT motif[2]	SignalP	TM domain	Highly variable in laboratory strains
					Le Roch et al [b]	Bozdech et al [c]					
PF07_0046	2655094	50S ribosomal protein L1, putative	3	28	Ring / Trophozoite	0	No	No	No	0	No
PFB0355c	812671	Cysteine protease, putative	10	95	Schizont	0	No	No	No	0	No

Footnotes

[1] Marti et al. [41]

[2] Hiller et al. [42]

TM domain - Number of transmembrane domains as predicted by the TMHMM2 algorithm.

The life cycle expression data is derived from Le Roch et al. 2003 [14] [b] and Bozdech et al. 2003 [25] [c]; 0 indicates a lower than 2-fold induction of expression at > = 50th percentile or no information. Gene IDs were derived from Entrez Gene at NCBI (http://www.ncbi.nlm.nih.gov/entrez/query.fcgi?db=gene).
