# Supplementary material for: A Systematic Map of Genetic Variation in Plasmodium falciparum
Source: PLoS Pathog. 2006 Jun 23;2(6):e57. doi: 10.1371/journal.ppat.0020057 (PMC1480597; doi:10.1371/journal.ppat.0020057)
Supplement: Supplementary file 7 [file ppat.0020057.st009.pdf]

Supplemental Table S9 Variation in characterized parasite immunogens in clinical development. Candidate parasite immunogens were derived from Richie and Saul, 2002 [2]

				Strain												
PlasmoDB_ID	Gene_ID	Description	Total number of probes	7G8	D6	W2	HB3	Dd2	FCR3	FCB	C188	18.02	31.01	41.02	48.02	51.02
PF11_0344	810891	Apical membrane antigen 1	65	8	5	8	10	8	9	7	8	4	4	4	4	10
PFC0210c	814364	Circumsporozoite protein	25	1	0	1	2	1	3	3	2	2	1	2	2	1
PF10_0344	810501	Glutamate rich protein	110	2	0	1	3	1	2	2	1	0	2	2	2	5
PFB0300c	812660	Merozoite surface protein 2	40	0	6	12	12	12	2	2	12	11	8	2	0	11
PFI1475w	813575	Merozoite surface protein 1	162	16	5	16	95	17	94	93	13	7	14	8	6	9
PFA0110w	813159	Ring infected erythrocyte surface antigen	79	0	0	1	0	0	0	0	0	0	0	0	1	0
PFB0915w	812783	Liver stage antigen 3	136	2	2	3	3	3	3	3	3	1	1	4	4	4
PF10_0356	810513	Liver stage antigen 1	55	2	1	9	8	8	2	4	8	1	13	8	2	14
PF13_0201	814170	Sporozoite surface protein 2	61	3	2	4	5	4	1	2	4	4	5	8	4	6
PF10_0345	810502	Merozoite surface protein 3	31	1	0	4	1	8	9	9	8	6	0	0	10	0
PFB0340c	812668	Serine rich antigen	100	2	1	6	2	7	4	5	7	5	3	6	6	7
PF11_0224	810771	Circumsporozoite-related antigen	18	0	0	0	0	0	0	0	0	0	2	0	0	0
		Total	882	37	22	65	141	69	129	130	66	41	53	44	41	67

Footnote
Gene IDs were derived from Entrez Gene at NCBI (http://www.ncbi.nlm.nih.gov/entrez/query.fcgi?db=gene).
