# Supplementary material for: A Systematic Map of Genetic Variation in Plasmodium falciparum
Source: PLoS Pathog. 2006 Jun 23;2(6):e57. doi: 10.1371/journal.ppat.0020057 (PMC1480597; doi:10.1371/journal.ppat.0020057)
Supplement: Supplementary file 8 [file ppat.0020057.st010.pdf]

Supplemental Table S10 The Log2 (ratio) and copy number, for all *P. falciparum* strains, of the GTP-cyclohydrolase I (PFL1155w), the two flanking genes (PFL1150c and PFL1160w), as well as an uncharacterized control gene (PFL0650c), also located on chromosome 12. The ratio and copy number of the *Plasmodium falciparum* multi drug resistance protein, *pfmdr1* (PFE1150w) and the *P. falciparum* 11-1 protein (PF10_0374) are also represented. A ratio histogram of the Log2 (ratio) for the HB3 / 3D7 signal intensity (**Figure S1**) verifies the thresholds used to identify gene amplifications. The GTP-cyclohydrolase copy numbers were extrapolated based on 3 copies in 3D7, and gene pf 11-1 based on 2 copies.

	Gene											
	PFL1150c (Ribosomal protein L24)		PFL1155w (GTP cyclohydrolase)		PFL1160w (uncharacterized protein		PFL0650c (uncharacterized protein)		PFE1150w (multi-drug resistance protein)		PF10_0374 (pf 11-1 protein)	
	Log2 (ratio)	Copy number	Log2 (ratio)	Copy number	Log2 (ratio)	Copy number	Log2 (ratio)	Copy number	Log2 (ratio)	Copy number	Log2 (ratio)	Copy number
FCR3	-0.09	0.94	-1.59	1.00	-0.24	0.84	-0.13	0.91	0.69	1.61	-1.05	0.96
HB3	0.92	1.89	-0.52	2.09	0.76	1.69	-0.09	0.94	-0.07	0.95	-0.23	1.71
D6	0.30	1.23	-0.46	2.18	0.07	1.05	0.12	1.09	-0.11	0.93	-0.55	1.37
W2	1.00	2.00	-0.43	2.23	-0.10	0.93	0.20	1.15	-0.09	0.94	-0.57	1.35
7G8	0.93	1.90	-0.41	2.26	1.06	2.08	-0.07	0.96	-0.09	0.94	-0.92	1.06
Dd2	0.50	1.41	-0.86	1.65	-0.42	0.75	0.05	1.04	0.75	1.69	-0.61	1.31
31.01	-0.01	1.00	-1.85	0.83	-0.05	0.97	0.06	1.04	-0.07	0.95	-1.41	0.75
18.02	-0.03	0.98	-1.88	0.82	-0.14	0.91	0.03	1.02	-0.10	0.93	-2.03	0.49
51.02	-0.07	0.95	-1.74	0.90	0.00	1.00	0.04	1.03	-0.10	0.93	-1.28	0.82
41.02	0.13	1.10	-1.81	0.86	-0.16	0.89	0.07	1.05	-0.04	0.97	-0.66	1.26
48.02	-0.01	1.00	-1.64	0.96	-0.19	0.88	0.01	1.01	-0.02	0.98	-0.87	1.10
FCB	0.95	1.93	-0.71	1.84	0.79	1.73	0.09	1.07	0.60	1.51	-0.68	1.25
3D7		1.00		3.00		1.00		1.00		1.00		2.00
