# Supplementary material for: A Systematic Map of Genetic Variation in Plasmodium falciparum
Source: PLoS Pathog. 2006 Jun 23;2(6):e57. doi: 10.1371/journal.ppat.0020057 (PMC1480597; doi:10.1371/journal.ppat.0020057)
Supplement: Supplementary file 9 [file ppat.0020057.st011.pdf]

Supplemental Table S11 Threshold cycle (Ct) values derived from the real-time PCR amplification of the *P. falciparum* genes: PFL1155w, PFL1145w, PFL1150c and PFE1150w, along with a ß-tubulin control gene, PF10_0084. A parallel reaction was carried out for each primer pair, and the mean Ct value and standard deviation are presented.

	Experiment 1			Experiment 2		
Strain	PF10_0084	PFL1155w	PFE1150w	PF10_0084	PFL1145w	PFL1150c
3D7	12.34 ±0.028	9.84 ±0.098	11.765 ±0.077	12.485 ±0.318	12.74 ±0.282	12.43 ±0.282
W2	11.07 ±0.155	10.165 ±0.219	10.545 ±0.091	12.045 ±0.247	11.905 ±0.007	11.595 ±0.275
FCR3	10.57 ±0.042	10.02 ±0.282	9.11 ±0	11.135 ±0.403	11.25 ±0.282	11.36 ±0.282
FCB	14.5 ±0.268	12.14 ±0	12.465 ±0.148	13.385 ±0.049	11.765 ±0.431	11.525 ±0.530
HB3	11.62 ±0.254	10.135 ±0.205	10.865 ±0.332	11.705 ±0.190	11.05 ±0.028	10.81 ±0.155
D6	12.105 ±0.077	11.73 ±0.084	12.035 ±0.360	13.2 ±0.070	13.835 ±0.205	13.19 ±0.014
Dd2	12.12 ±0.070	10.915 ±0.205	9.85 ±0.014	11.57 ±0.070	11.61 ±0.367	10.94 ±0.141
7G8	12.365 ±0.120	10.41 ±0.014	11.85 ±0.084	12.01 ±0.141	11.265 ±0.007	10.555 ±0.148
18.02	12.31 ±0.042	12.17 ±0.042	12.075 ±0.091	12.82 ±0.014	13.3 ±0.155	12.98 ±0.042
31.01	11.92 ±0	11.815 ±0.007	11.605 ±0.091	12.38 ±0.311	12.625 ±0.134	12.39 ±0.296
41.02	12.86 ±0.014	12.9 ±0.028	12.635 ±0.035	13.4 ±0.141	13.78 ±0.056	13.41 ±0.042
48.02	12.81 ±0.056	12.98 ±0.028	12.7 ±0.028	13.485 ±0.219	13.995 ±0.360	13.67 ±0.197
51.02	12.93 ±0	12.94 ±0.127	12.615 ±0.021	13.9 ±0.113	14.31 ±0.155	14.095 ±0.049
