# Supplementary material for: A Systematic Map of Genetic Variation in Plasmodium falciparum
Source: PLoS Pathog. 2006 Jun 23;2(6):e57. doi: 10.1371/journal.ppat.0020057 (PMC1480597; doi:10.1371/journal.ppat.0020057)
Supplement: Supplementary file 10 [file ppat.0020057.st013.pdf]

Supplemental Table S13 Description of the genes deleted in the *P. falciparum* strains analyzed; Deletions are only reported for genes with ≥ six probes. No deletions in strain D6 were observed.

PlasmoDB_ID	Gene_ID	Description	Strain												
			18.02	31.01	41.02	48.02	51.02	3D7	7G8	C188	Dd2	FCB	FCR3	HB3	W2
PF10_0007	810166	Uncharacterized protein										1			
PF10_0008	810167	Uncharacterized protein										1	1		
PF10_0014	810172	Uncharacterized protein								1	1				1
PF10_0343	810500	S-antigen		1			1		1					1	
PF11_0012	810580	Uncharacterized protein				1			1	1	1	1	1	1	1
PF11_0014	810578	Uncharacterized protein (maurers cleft protein)												1	
PF13_0115	814086	Frameshifted ebl1, pseudogene												1	
PF14_0732	812314	Uncharacterized protein				1	1			1				1	
PF14_0733	812315	Uncharacterized protein				1	1			1				1	
PF14_0734	812316	Uncharacterized protein				1	1			1				1	
PFA0065w	813150	Uncharacterized protein (maurers cleft protein)	1	1	1	1	1		1	1	1	1	1	1	1
PFB0070w	812610	Uncharacterized protein								1	1				1
PFB0075c	812611	Uncharacterized protein								1	1				1
PFB0080c	812612	Uncharacterized protein								1	1				1
PFB0085c	812613	Uncharacterized protein								1	1				1
PFB0090c	812614	Uncharacterized protein, conserved								1	1				
PFB0095c	812615	Erythrocyte membrane protein 3								1	1				
PFB0965c	812797	Uncharacterized protein												1	
PFB0970c	812798	Uncharacterized protein												1	
PFC0045w	814327	Uncharacterized protein								1				1	
PFC1080c	814556	Uncharacterized protein (maurers cleft protein)					1								

PlasmoDB_ID	Gene_ID	Description	Strain												
			18.02	31.01	41.02	48.02	51.02	3D7	7G8	C188	Dd2	FCB	FCR3	HB3	W2
PFD1205w	812411	Predicted integral membrane protein		1											
PFD1210w	812412	Uncharacterized protein		1											
PFI1475w	813575	Merozoite surface protein 1, precursor*												1	
PFI1710w	813622	Cytoadherence-linked protein										1	1		
PFI1785w	813637	Uncharacterized protein										1	1		
PFI1800w	813640	Enzyme, putative										1	1		
PFL2360w	811524	Uncharacterized protein						1				1			
PFL2550w	811560	Uncharacterized protein, conserved in P. falciparum									1				
PFL2555w	811561	Uncharacterized protein									1				
PFL2565w	811563	Uncharacterized protein												1	
PFL2570w	811564	Acyl-coa ligase antigen												1	
PFL2580w	811566	Uncharacterized protein		1			1				1				1

Footnotes

* Visual examination of the hybridization pattern of MSP-1 indicates a partial deletion or substitution.

Gene IDs were derived from Entrez Gene at NCBI (http://www.ncbi.nlm.nih.gov/entrez/query.fcgi?db=gene).
