# Supplementary material for: A Systematic Map of Genetic Variation in Plasmodium falciparum
Source: PLoS Pathog. 2006 Jun 23;2(6):e57. doi: 10.1371/journal.ppat.0020057 (PMC1480597; doi:10.1371/journal.ppat.0020057)
Supplement: Supplementary file 11 [file ppat.0020057.st014.pdf]

Supplemental Table S14 Oligonucleotide primer sequences

PlasmoDB_ID	Primer	Direction	Sequence 5' – 3'
PF10_0348	348-1	Forward	aaaaatatcaaatgcaatgacctaa
	348-2	Reverse	tccttgtagaggagaataaccagaa
	348-3	Forward	ttgaagaacatgaaaataaatcttacc
	348-4	Reverse	taaaatttttctcgtctttgattcg
	348-5	Forward	gtagaaatggggattggattagtgt
	348-6	Reverse	cttcccaccagtctacacgtttt
	348-7	Forward	aatatggattttcagatatgggagat
	348-8	Reverse	ttttcagcagttatacttttagtatcttc
	348-9	Forward	agcgaatataaaaataagtttgaggatg
	348-10	Reverse	ttgagatgttgttatattccctcgtt
	348-11	Forward	caaccacttctttatcaacgtctatt
	348-12	Reverse	gtttcttctccttcattttccaattc
	348-13	Forward	tctctagaattaataaaattaacatcaaagg
	348-14	Reverse	aaatctgtcatatcttctgtcaaacc
PFE1600w	1600-1	Forward	aatggatcaatttatatttctcctag
	1600-2	Reverse	tggaatcttgtccacatgtcatcac
	1600-3	Forward	tcaaaatgttgtgattctataagtg
	1600-4	Reverse	agcattgtcttcagtttccttt
	1600-5	Forward	atgtgaacttgatgacaatagag
	1600-6	Reverse	aaaggagggtatgatttaccaaatac
	1600-7	Forward	cgcatgaaaatacctacccaac
	1600-8	Reverse	ttcaccattttcatcttcttgc
PFL0800c	0800-1	Forward	ctcatattttgatttcattttatgttaatg
	0800-2	Reverse	caccaaccttttttagaatcaagc
	0800-3	Forward	ctgggtcgacttttggtggttt
	0800-4	Reverse	gtcattctccttattatatatacctttttgg
